# A Class 1 Histone Deacetylase with Potential as an Antifungal Target

**DOI:** 10.1128/mBio.00831-16

**Published:** 2016-11-01

**Authors:** Ingo Bauer, Divyavaradhi Varadarajan, Angelo Pidroni, Silke Gross, Stefan Vergeiner, Birgit Faber, Martin Hermann, Martin Tribus, Gerald Brosch, Stefan Graessle

**Affiliations:** a Division of Molecular Biology, Biocenter, Medical University of Innsbruck, Innsbruck, Austria; b Department of Anesthesiology and Intensive Care Medicine, Medical University of Innsbruck, Innsbruck, Austria

## Abstract

Histone deacetylases (HDACs) remove acetyl moieties from lysine residues at histone tails and nuclear regulatory proteins and thus significantly impact chromatin remodeling and transcriptional regulation in eukaryotes. In recent years, HDACs of filamentous fungi were found to be decisive regulators of genes involved in pathogenicity and the production of important fungal metabolites such as antibiotics and toxins. Here we present proof that one of these enzymes, the class 1 type HDAC RpdA, is of vital importance for the opportunistic human pathogen *Aspergillus fumigatus*. Recombinant expression of inactivated RpdA shows that loss of catalytic activity is responsible for the lethal phenotype of *Aspergillus* RpdA null mutants. Furthermore, we demonstrate that a fungus-specific C-terminal region of only a few acidic amino acids is required for both the nuclear localization and catalytic activity of the enzyme in the model organism *Aspergillus nidulans*. Since strains with single or multiple deletions of other classical HDACs revealed no or only moderate growth deficiencies, it is highly probable that the significant delay of germination and the growth defects observed in strains growing under the HDAC inhibitor trichostatin A are caused primarily by inhibition of catalytic RpdA activity. Indeed, even at low nanomolar concentrations of the inhibitor, the catalytic activity of purified RpdA is considerably diminished. Considering these results, RpdA with its fungus-specific motif represents a promising target for novel HDAC inhibitors that, in addition to their increasing impact as anticancer drugs, might gain in importance as antifungals against life-threatening invasive infections, apart from or in combination with classical antifungal therapy regimes.

## IMPORTANCE

This paper reports on the fungal histone deacetylase RpdA and its importance for the viability of the fungal pathogen *Aspergillus fumigatus* and other filamentous fungi, a finding that is without precedent in other eukaryotic pathogens. Our data clearly indicate that loss of RpdA activity, as well as depletion of the enzyme in the nucleus, results in lethality of the corresponding *Aspergillus* mutants. Interestingly, both catalytic activity and proper cellular localization depend on the presence of an acidic motif within the C terminus of RpdA-type enzymes of filamentous fungi that is missing from the homologous proteins of yeasts and higher eukaryotes. The pivotal role, together with the fungus-specific features, turns RpdA into a promising antifungal target of histone deacetylase inhibitors, a class of molecules that is successfully used for the treatment of certain types of cancer. Indeed, some of these inhibitors significantly delay the germination and growth of different filamentous fungi via inhibition of RpdA*.* Upcoming analyses of clinically approved and novel inhibitors will elucidate their therapeutic potential as new agents for the therapy of invasive fungal infections—an interesting aspect in light of the rising resistance of fungal pathogens to conventional therapies.

## INTRODUCTION

In addition to distinct regulatory sequences in gene promoters, the readout of genetic information in eukaryotes is significantly controlled at the chromatin level (1). Chromatin is the final structural result of various processes and phenomena around its building blocks, the nucleosome core particles, and changes in chromatin structure lead to short- and long-term alterations of the transcriptional activity of genes. The dynamics of chromatin are affected not only by intrinsic cellular programs but also by extrinsic factors in the environment. Consequently, chromatin dynamics play a crucial role in metabolism, development, and differentiation, as well as in the development of disease (e.g., references 2 and 3).

Besides ATP-dependent chromatin remodeling and DNA methylation, covalent posttranslational modifications of histones have significant structural and functional consequences for chromatin architecture (for a review, see reference 4). Most of these modifications occur on specific amino acids clustered in the N-terminal tails of core histones (5) and contribute to the modulation of DNA repair, replication, or transcription by tuning the accessibility of DNA for a multitude of regulatory factors (6–8).

To maintain the flexibility of the cell to adapt to changing exogenous conditions, histone modifications have to be reversible. Antagonistic enzymes ensure a delicate equilibrium of modified and nonmodified residues of the core histones. One prominent example of such a subtle balance is the reversible acetylation of distinct lysine residues by histone acetyltransferases (HATs) and histone deacetylases (HDACs) (for a review, see reference 9).

Irrespectively of their specific mode of action, hyperacetylated histones are usually associated with transcriptionally active genomic regions, whereas deacetylation is linked to repression and silencing. In concert with other modifications, however, not only does acetylation act as a specific signal for the recruitment of distinct transcription factors (10–12), but in fact, those factors themselves might be substrates of HATs and HDACs (13–15). Since disorders in the acetylation pattern lead to transcriptional deregulation, the activity of these enzymes is also correlated with the development of some tumors in humans. Hence, several natural and synthetic inhibitors of classical HDACs are already in use or are under evaluation in clinical trials against different types of cancer (16, 17). Several of these inhibitors show only little effect on normal tissues, and some of them are even specific for distinct HDAC classes (18, 19).

In higher eukaryotes, classical HDACs can be divided into at least three classes with more than 10 different types of enzymes. In contrast, fungi possess only four to six members of the classical HDAC family (20, 21). In *Aspergillus nidulans* and its pathogenic relatives, two class 1 enzymes, RpdA and HosA (22, 23), and two class 2 HDACs, HdaA and HosB, were identified and characterized (24) (see Fig. S1 in the supplemental material). *Aspergillus* strains lacking class 2-type enzymes showed several deficiencies, including hypersensitivity to stress conditions, affected germination (25–28), and most notably, significant deregulation of the production of important bioactive molecules with deleterious (e.g., toxins) and beneficial (e.g., antibiotics) properties (29, 30).

Whereas class 2 HDAC deletion mutants were all viable, several efforts to generate an *Aspergillus* RpdA minus strain failed. This led to the hypothesis that, in contrast to *Saccharomyces cerevisiae*, RpdA-type enzymes might play an essential role in filamentous fungi. This assumption was confirmed recently for the model organism *A. nidulans* by the expression of RpdA under the control of the alcohol dehydrogenase (*alcA*) and xylanase (*xylP*) promoters, respectively (31). By combining these promoters into a conditional two-promoter system, we were able to prove that a C-terminal region of approximately 70 amino residues (C70) of RpdA cannot be deleted without affecting the vitality of the fungus (31).

Now we demonstrate that this essential C-terminal part of RpdA can be pinpointed to a highly charged motif of only a few acidic residues unique in HDACs of filamentous fungi. Since this motif is required for both nuclear localization and catalytic activity of RpdA, homologous enzymes of other filamentous fungi, including those of the pathogenic species *Aspergillus fumigatus* and *Cochliobolus carbonum*, but not those of higher eukaryotes, are able to complement *A. nidulans* strains depleted of native RpdA activity.

The vital importance of RpdA turns this enzyme into a promising target for HDAC inhibitors (HDACIs) with antifungal activity and might extend their current use from drugs against certain types of cancer to substances administered for the treatment of fungal infections. Indeed, the germination, growth, and conidiation of *A. fumigatus* and other fungal species are significantly delayed when the HDACI trichostatin A (TSA) is added to the medium.

## RESULTS

### RpdA, an essential enzyme for *A. fumigatus*.

**FIG 1:**
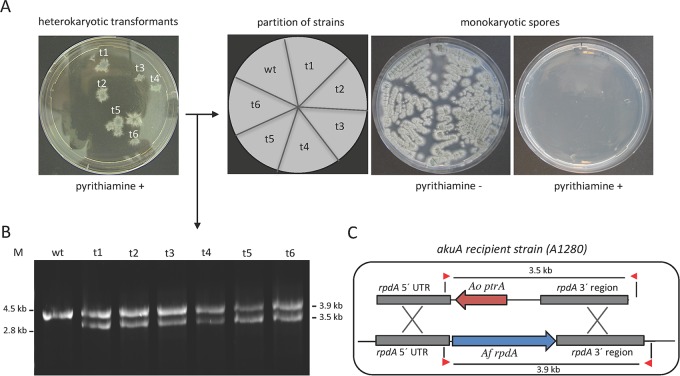
Heterokaryon rescue of an *A. fumigatus* Ku70 recipient strain transformed with an *rpdA* deletion cassette. After transformation, heterokaryotic strains t1 to t6 were grown under selective conditions (pyrithiamine) and uninucleate conidia were streaked onto sectors on agar plates with and without selection and grown for 48 h at 37°C (A). Molecular size markers (M) are shown on the left and right. Genomic DNA from putative heterokaryons was prepared and subjected to a diagnostic PCR. DNA of the recipient strain served as a control (B). The scheme of the homologous integration of the deletion cassette, the annealing sites of the primers, and the sizes of the fragments amplified are shown in panel C. *Ao ptrA*, pyrithiamine resistance gene of *A. oryzae*, *Af rpdA*, coding sequence of *A. fumigatus* RpdA. UTR, untranslated region; wt, wild type.

A future application of HDACIs as antifungal drugs implies that, in addition to *A. nidulans*, RpdA is also essential for other fungal species. In order to investigate the impact of RpdA for the most frequent cause of invasive fungal infections in immunocompromised patients, *A. fumigatus*, the heterokaryon rescue technique was used. This method takes advantage of a feature common to many filamentous fungi to produce uninucleate conidia while maintaining multinucleate hyphae (32). *A. fumigatus* strain A1280, an *akuA* mutant minimizing heterologous integrations of DNA, was used as the recipient of an *rpdA* deletion cassette comprising the pyrithiamine resistance gene *ptrA* (Fig. 1C). Transformants were recovered under selective growth conditions, leading to strains with wild-type nuclei (providing RpdA activity to the cells) and Δ*rpdA* mutant nuclei (providing the pyrithiamine resistance). Subsequently, mononuclear conidia of the recovered colonies and the recipient strain were streaked onto minimal medium (MM)-agar plates with and without selection. In contrast to plates without pyrithiamine, where all strains were able to grow, none of the conidia germinated under selective conditions (Fig. 1A). This indicated that all of the transformants analyzed were heterokaryotic, comprising nuclei of the genotypes *ptrA^−^/rpdA^+^* and *ptrA^+^/rpdA*^−^, respectively, and strongly suggested that RpdA is crucial for the viability of *A. fumigatus*. For verification, genomic DNA was prepared from transformants and used as a template for an analytical PCR approach with primers specific for the 5′- and 3′-untranslated regions of *rpdA* (Fig. 1C). Whereas the recipient yielded a PCR product of 3.9 kb (representing the coding sequence of *rpdA*), two fragments were amplified from DNA of the mutant strains (Fig. 1B): the *rpdA* wild-type fragment (3.9 kb) and one product comprising the *ptrA* marker gene integrated at the *rpdA* locus (3.5 kb). This screening confirmed that all of the transformants analyzed were heterokaryotic and proved that RpdA is, in fact, essential for *A. fumigatus*.

### Catalytic activity of RpdA is essential for growth and development of *A. nidulans*.

**FIG 2:**
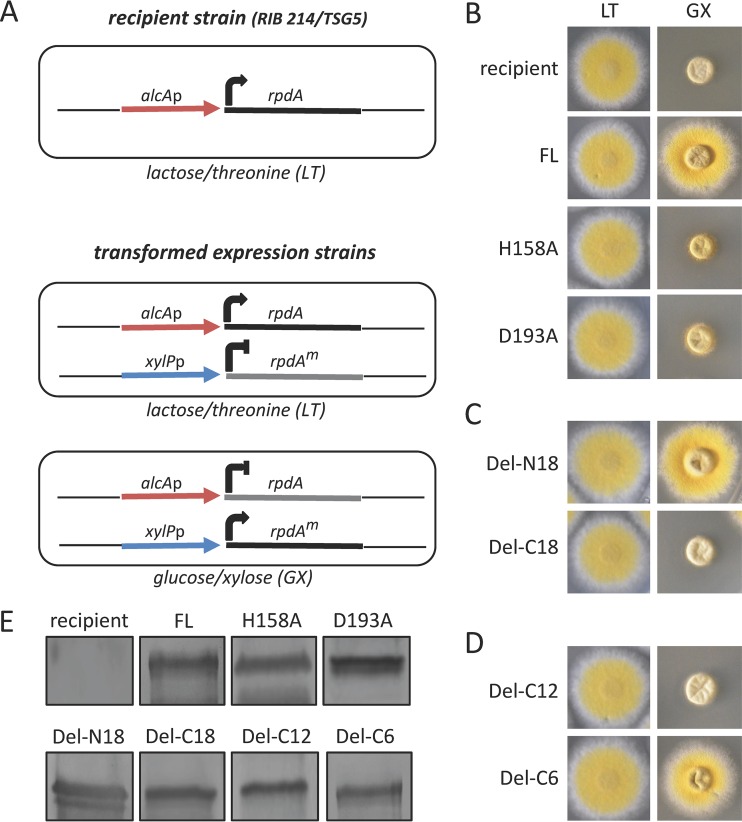
Phenotypic analysis of *Aspergillus* strains expressing different RpdA variants. A two-promoter system was used to determine the biological function of mutated RpdA fragments. Recipient strains RIB214 and TSG5 expressing endogenous *rpdA* (full length, FL) under the control of the alcohol dehydrogenase promoter (*alcAp*) were transformed with expression cassettes comprising the coding regions of different RpdA variants under the control of the xylanase promoter (*xylPp*) (A). Transformed protoplasts were regenerated under *alcAp* induction (LT), and RpdA variants H158A and D193A (B), del-N18 and del-C18 (C), and del-C12 and del-C6 (D) were analyzed for the ability to compensate for wild-type RpdA depletion under *alcAp* repressive and *xylPp* inductive conditions (GX). Expression of the recombinant variants was verified by immunoblotting of whole protein extracts under GX conditions with anti-RpdA antibodies. Recipient strain TSG5 (no *xylPp* expression cassette) and a strain expressing wild-type RpdA under the control of *xylPp* (FL) were used as negative and positive controls, respectively (E).

Like many class 1 type HDACs, RpdA is enzymatically active as part of large multiprotein complexes (e.g., see reference 24) and the composition of several of these complexes was elucidated in yeast and higher eukaryotes (e.g., see references [Bibr B33][Bibr B34][Bibr B37]). For two of these complexes in yeast, a previously unrecognized function as a histone chaperone and a chromatin-stabilizing factor was recently suggested, leading to transcriptional repression irrespective of the catalytic activity of the complexes (38). In order to determine whether the growth retardation of *Aspergillus* rpdA null mutants is due to the lack of chromatin stabilization function or due to loss of enzymatic RpdA activity, we examined the phenotypes of two strains expressing RpdA with mutations of N-terminal residues known to be essential for HDAC activity (see Fig. S2B in the supplemental material) (39). The expression construct of the first strain led to the production of RpdA with a mutation of histidine 158 to alanine (H158A), and in the second construct, aspartate 193 was substituted for alanine (D193A). Both proteins were expressed in *A. nidulans* strain TSG5 with a two-promoter expression system described elsewhere (31). TSG5, which holds endogenous RpdA under the control of the alcohol dehydrogenase promoter (*alcAp*), was transformed with an expression cassette comprising mutated *rpdA* under the control of the heterologous xylanase promoter (*xylPp*)*.* These promoters can be induced independently by the addition of lactose and threonine (LT) and glucose and xylose (GX), respectively (Fig. 2A). Moreover, for labeling of the nuclei (described later), a red fluorescent histone protein (H2A-mRFP) is constitutively expressed in this strain. TSG5 was transformed with the mutated RpdA fragments under the control of *xylPp*, and transformants were recovered under inducing conditions of wild-type RpdA (LT). Subsequently, strains were phenotypically analyzed under GX conditions. Although both mutated RpdA fragments were sufficiently expressed under GX (Fig. 2E), TSG1.16 (H158A) and TSG2.15 (D193A) resembled the lethal phenotype of the recipient TSG5 (Fig. 2B). This result confirms that the growth phenotype of RpdA-depleted *A. nidulans* is caused by the lack of catalytic HDAC activity.

### RpdA inhibition by TSA delays germination and arrests growth and conidiation of *A. fumigatus* and other fungal species.

**FIG 3:**
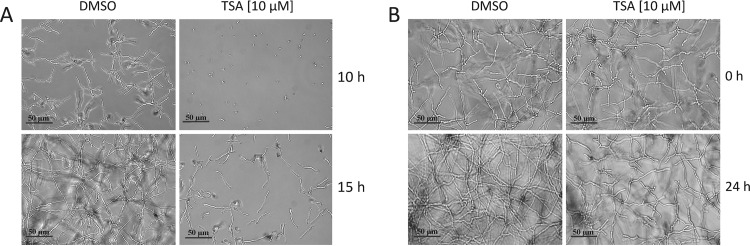
Germination of spores and hyphal growth of *A. fumigatus* under TSA treatment. Conidia (1 × 10^5^/ml) were incubated into 24-well plates with RPMI medium supplied with 10 µM TSA. Spores were incubated for 10 or 15 h at 37°C before wells were examined under a light microscope (A). Growth retardation of hyphae was observed in liquid medium 24 h after the addition of 10 µM TSA to a culture grown for 10 h at 37°C without an inhibitor (B). DMSO, the solvent of TSA, was used in the corresponding concentration as a negative control.

**FIG 4:**
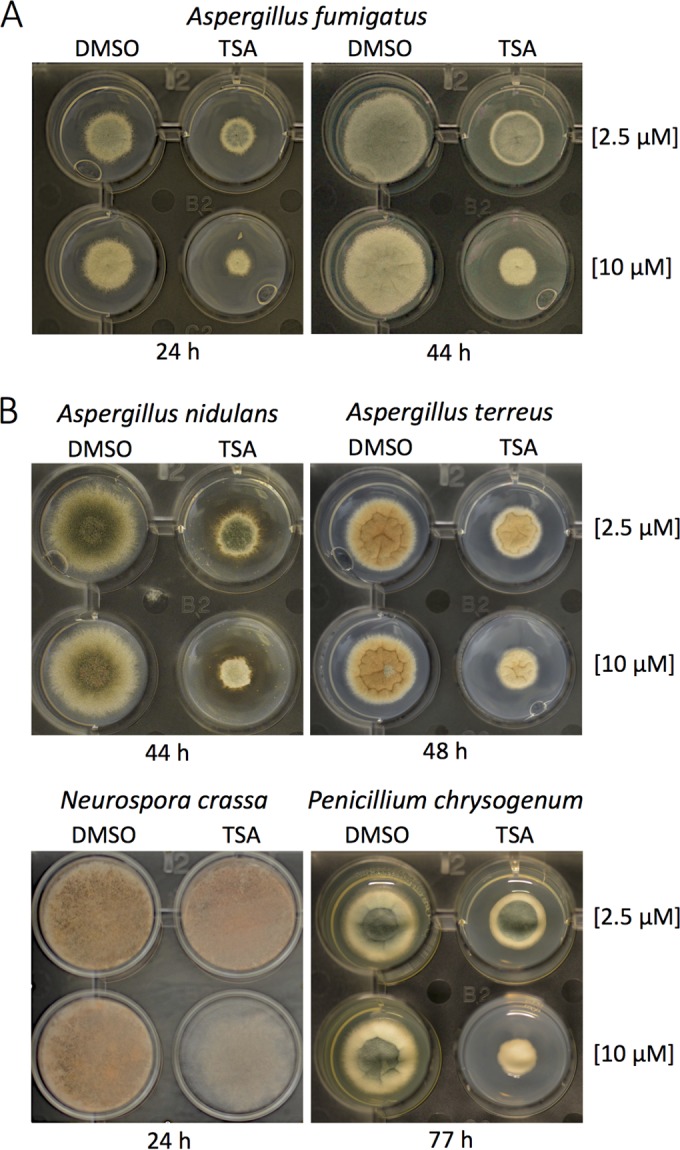
Mycelial growth and sporulation of *A. fumigatus* (A), *A. nidulans*, *A. terreus*, *N. crassa*, and *P. chrysogenum* (B) at different TSA concentrations. Spores (1 × 10^3^) were dotted into the middle of each agar well, and strains were grown overnight to allow germination. Subsequently, colonies were overlaid with 100 µl of liquid medium containing 2.5 or 10 µm of the inhibitor. A corresponding concentration of DMSO was used as a negative control. After incubation for different periods of time at 37°C (*A. fumigatus*, *A. nidulans*, *A. terreus*, and *N. crassa*) or 25°C (*P. chrysogenum*), the size of the colony and conidiation of the mycelium were assessed.

TSA, a metabolite produced by *Streptomyces* sp., is a potent inhibitor of catalytic activity of classical HDACs. Moreover, TSA was supposed to have potential as an anticancer drug (40). We have demonstrated that TSA is able to inhibit HDAC activity in crude protein extracts of *A. nidulans* *in vitro* in the nanomolar range (24). In order to pre-examine (i) the efficacy of TSA with regard to the specific inhibition of RpdA and (ii) TSA stability in cultures at 37°C used for subsequent inhibition assays *in vivo*, tandem affinity purification (TAP)-tagged RpdA was expressed in *A. nidulans*, affinity purified under native conditions as described below, and used in an HDAC activity assay with tritium-labeled chicken histones (24, 25). Catalytic activity was determined with different concentrations of TSA from a stock solution (dissolved in dimethyl sulfoxide [DMSO]) or retrieved from the supernatant of liquid *A. fumigatus* cultures grown in the presence of TSA for 0, 5, or 24 h at 37°C. Already 50 nM TSA sufficiently inhibited 70% of RpdA activity and, unexpectedly, even after 24 h at 37°C in the culture medium, no loss of inhibition was detectable (see Fig. S3 in the supplemental material). In view of a possible therapeutic application of HDACIs against fungal infections, we set out to investigate the effect of TSA with regard to the germination efficiency of *Aspergillus* spores. To this end, we inoculated conidia of *A. fumigatus* A1163 into liquid medium (RPMI) supplemented with 10 µM TSA and examined them under a light microscope after incubating them for 10 or 15 h at 37°C. In contrast to a DMSO control, no or only negligible germination occurred after 10 h. After 15 h, spores started to germinate; however, the number of germinating spores was significantly lower and hyphal length was remarkably shorter than those of a nontreated control (Fig. 3A). To test if vegetative growth of *A. fumigatus* is also affected by TSA, spores were incubated for 10 h at 37°C before the inhibitor was added to the germlings to a final concentration of 10 µM. Even 24 h after TSA addition, treated strains showed a significant retardation of growth and mycelia were considerably disordered and highly branched (Fig. 3B). In order to confirm the antifungal effect of TSA on solid medium, fungal cultures were overlaid with different concentrations of the inhibitor after germination and incubated for a further 24 or 44 h at 37°C. Even a 2.5 µM concentration of the inhibitor led to a reduced colony diameter at both time points, and at a 10 µM concentration, growth was significantly arrested and conidiation was considerably inhibited (Fig. 4A). Since earlier investigations have shown that single and even combined deletions of the three remaining HDACs, HdaA, HosA, and HosB, did not lead to comparable defects in germination, growth, or development (25, 26, 31), it is justified to speculate that the phenotype caused by TSA treatment is primarily due to inhibition of RpdA activity.

The significant effects of TSA led to the question of whether other HDACIs show comparable efficacy against RpdA. To address this issue, vorinostat (suberanilohydroxamic acid [SAHA]) and apicidin were tested for RpdA inhibition as well. SAHA was the first HDACI to be approved for the treatment of certain types of cancer (41), and also the fungal metabolite apicidin has been reported as an HDACI with an antitumor effect (42). Both inhibitors were used in concentrations of 50 and 500 nM in our HDAC assay with the affinity-purified RpdA activity. Whereas SAHA showed only weak inhibition of RpdA, the efficacy of apicidin was significantly higher but also did not reach the inhibitory effect of TSA (see Fig. S4A in the supplemental material). These differences are also in line with the subsequently performed growth assays with *A. fumigatus* (see Fig. S4B). Two hundred fifty micromolar SAHA inhibited mycelial growth to approximately the same extent as 25 µM apicidin, reflecting the about 10-fold higher efficacy of apicidin against RpdA in the HDAC assay (see Fig. S4A in the supplemental material). However, even 50 µM apicidin did not reach the level of inhibition observed with 10 µM TSA (Fig. 4A).

In order to strengthen our assumption that inhibition of RpdA-type enzymes by HDACIs might affect the growth and development of many (if not all) filamentous fungi, we also tested other fungal species for inhibition by TSA. Spores of *A. nidulans*, its pathogenic relative *A. terreus* (43), and those of two distantly related fungi, *Penicillium chrysogenum* and *Neurospora crassa*, were dotted onto solid medium supplemented with different TSA concentrations as described above. Plates were incubated for the appropriate times at the appropriate growth temperatures before colony sizes and sporulation were assessed. As shown in Fig. 4B, both growth and conidiation were inhibited to very similar extents, as was observed with *A. fumigatus* (Fig. 4A), suggesting that the viability of many filamentous fungi can be considerably restricted by inhibition of catalytic RpdA activity*.* The significance of RpdA-type enzymes prompted us to look more closely at the fungus-specific features of this interesting group of fungal HDACs.

### A fungus-specific C-terminal motif of 12 amino acids is required for RpdA function.

**FIG 5:**
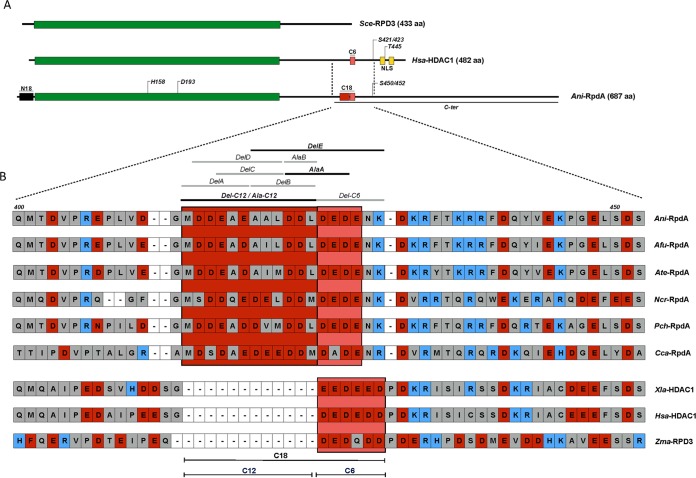
Comparison of RPD3-type HDACs of fungi and higher eukaryotes. A schematic representation of *S. cerevisiae* (*Sce*) RPD3, *Homo sapiens* (*Hsa*) HDAC1, and *A. nidulans* (*Ani*) RpdA is shown*.* The highly conserved region comprising amino residues essential for catalytic activity is green, the N-terminal fungus-specific region (N18) and the acidic C-terminal stretch conserved in filamentous fungi and higher eukaryotes (C18 and C6) are black and red, respectively. Putative nuclear localization sequences in enzymes of higher eukaryotes are yellow. The C-terminal tail (C-ter) expressed as a Venus-tagged peptide is indicated (A). aa, amino acids. A detailed alignment of the region adjacent to the acidic C-terminal motif essential for RpdA-type enzymes of filamentous fungi is shown for different fungal species, amphibians (*Xenopus*), humans, and plants (*Zea*) in panel B. Stretches conserved in filamentous fungi (C12) and in all eukaryotes except yeasts (C6) are shown as black lines at the bottom. Residues are shaded red (acidic), blue (basic), or gray (uncharged). Deletions or alanine substitutions of the RpdA variants tested are shown at the top. Gray lines represent mutations with no effect on the biological function of RpdA, and black lines depict mutations leading to a lethal phenotype of the corresponding expression strains. *Afu*, *A. fumigatus*; *Ate*, *A. terreus*; *Ncr*, *N. crassa*; *Pch*, *P. chrysogenum*; *Cca*, *C. carbonum*; *Xla*, *Xenopus laevis*; *Zma*, *Zea mays*; *Hsa*, *Homo sapiens*.

Earlier investigations revealed that the size of RpdA-type HDACs in filamentous fungi considerably exceeds that of homologous enzymes in other eukaryotes, mainly because of an extension of the RpdA C terminus (31). In order to elucidate fungus-specific functions of RpdA-type enzymes, sequence alignments with RPD3-type HDACs of mammals, amphibians, plants, and fungi were performed. These analyses revealed that, in addition to the highly conserved catalytic domain present in all classical HDACs, RpdA-type enzymes of filamentous fungi possess two remarkable regions, each approximately 18 residues in length: (i) an extension at the N-terminal end (N18; see Fig. S2A in the supplemental material) and (ii) a motif within the extended C terminus (C18; Fig. 5).

The high conservation of these two fungus-specific regions prompted us to investigate their relevance for the biological function of the enzyme. To this end, RpdA with N18 or C18 deleted was expressed under the control of the *xylP* promoter in strain RIB214, again applying the two-promoter system described for strain TSG5. Although both truncated RpdA fragments were sufficiently expressed, only RpdA-ΔN18 was able to substitute for the repressed full-length enzyme (Fig. 2C and E). This result strongly suggests that only the conserved C-terminal stretch is required for the functional activity of the fungal enzyme. It is important to mention that C18 is part of a C-terminal region of approximately 70 amino acids that was already previously supposed to be crucial for the biological function of the enzyme (31). The obvious impact of C18 prompted us to reassess the multiple sequence alignments of the C termini of RPD3-type enzymes of filamentous fungi and those of higher eukaryotes in detail. This analysis revealed that one part within the essential C18 region is exclusively conserved in fungal HDACs, while another part is present in enzymes of higher eukaryotes as well. More precisely, C18 contains 12 fungus-specific acidic residues (C12) and 4 to 6 further acidic amino acids (C6) that are also conserved in enzymes of mammals and plants (Fig. 5B). To further examine the functional importance of these two stretches, RpdA was expressed with the corresponding deletions of C12 or C6 in RIB214. Interestingly, the strain expressing RpdA-ΔC6 was able to grow under *alcAp*^−^/*xylPp*^+^ conditions, whereas the RpdA-ΔC12-expressing strain resembled the sick phenotype of the recipient (Fig. 2D and E).

This rather surprising result indicated that the short stretch of acidic amino acids conserved in class 1 enzymes of higher eukaryotes obviously is not required for phenotypic complementation, whereas the fungus-specific motif of 12 mostly negatively charged residues cannot be deleted without affecting the biological function of RpdA—an exciting aspect in view of the importance of this enzyme for *A. nidulans* and its pathogenic relatives *A. fumigatus* and *A. terreus*.

### Human HDAC1 is not able to substitute for *A. nidulans* RpdA.

**FIG 6:**
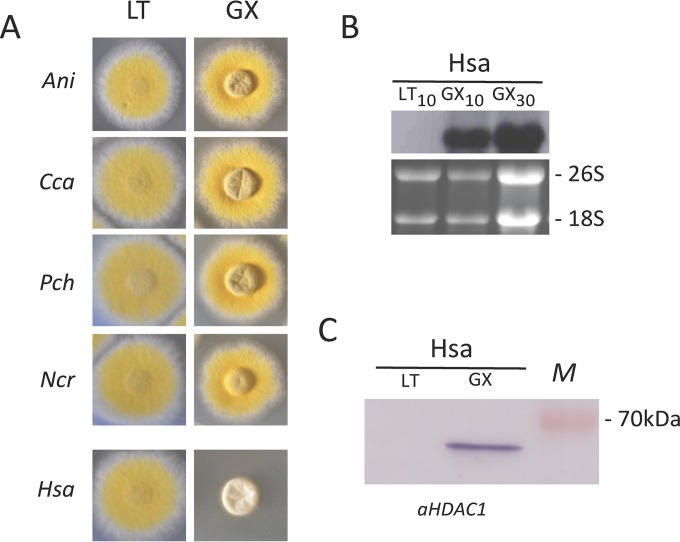
(A) Phenotypic analysis of *Aspergillus* strains expressing RpdA-type enzymes of different filamentous fungi and human HDAC1. Strains TSG5 and RIB214 expressing nonmutated RpdA under the control of the alcohol dehydrogenase promoter (*alcAp*) (Fig. 2A) were transformed with expression cassettes comprising the coding regions of the different RPD3-type HDACs under the control of the xylanase promoter (*xylPp*). Transformed protoplasts were regenerated under *alcAp* induction (LT), and recombinant heterologous HDACs were analyzed for the ability to compensate for RpdA under *alcAp* repressive and *xylPp* inductive conditions (GX). A strain of *A. nidulans* (*Ani*) expressing wild-type RpdA under the control of *xylPp* was used as a positive control. (B) Transcription of noncomplementing *HDAC1* (*H. sapiens* [*Hsa*]) was verified by Northern analysis with 10 and 30 µg of total RNA. rRNA was used as a loading and quality control. (C) Expression of HDAC1 was further confirmed by immunoblotting. Crude protein extract of *Hsa* grown under HDAC1 inductive (GX) conditions was blotted and probed with an anti-HDAC1 antibody. A 70-kDa marker protein (lane M) is shown. As a negative control for both Northern and Western analyses, the corresponding strains were grown under *xylPp* repressive conditions (LT). *Cca*, *C. carbonum*; *Pch*, *P. chrysogenum*; *Ncr*, *N. crassa*.

In order to assess whether RPD3-type HDACs of other species are able to complement RpdA, the human RpdA homolog HDAC1 and those of three filamentous fungi, *N. crassa*, *P. chrysogenum*, and *C. carbonum*, were expressed in *A. nidulans* strain RIB214 (HDAC1) or TSG5 (fungal HDACs) with the two-promoter system (Fig. 2A). Although only distantly related to each other, all of the fungal HDACs have the conserved C12 motif within an otherwise variable C terminus. As expected, all of the transformants resembled the wild-type phenotypes under *alcAp* inductive conditions because of the expression of endogenous RpdA. Under *xylPp* induction, however, only fungal orthologs comprising C12 were able to compensate for RpdA depletion (Fig. 6A), although also human HDAC1 was clearly expressed, as shown by Northern analysis and immunoblotting (Fig. 6B and C). This result further confirmed the assumption that the fungus-specific acidic C-terminal stretch might contribute to the functional activity of RPD3-type enzymes in filamentous fungi and prompted us to look for anomalies in the ΔC12 strains.

### The fungus-specific acidic region is required for the nuclear localization of RpdA.

**FIG 7:**
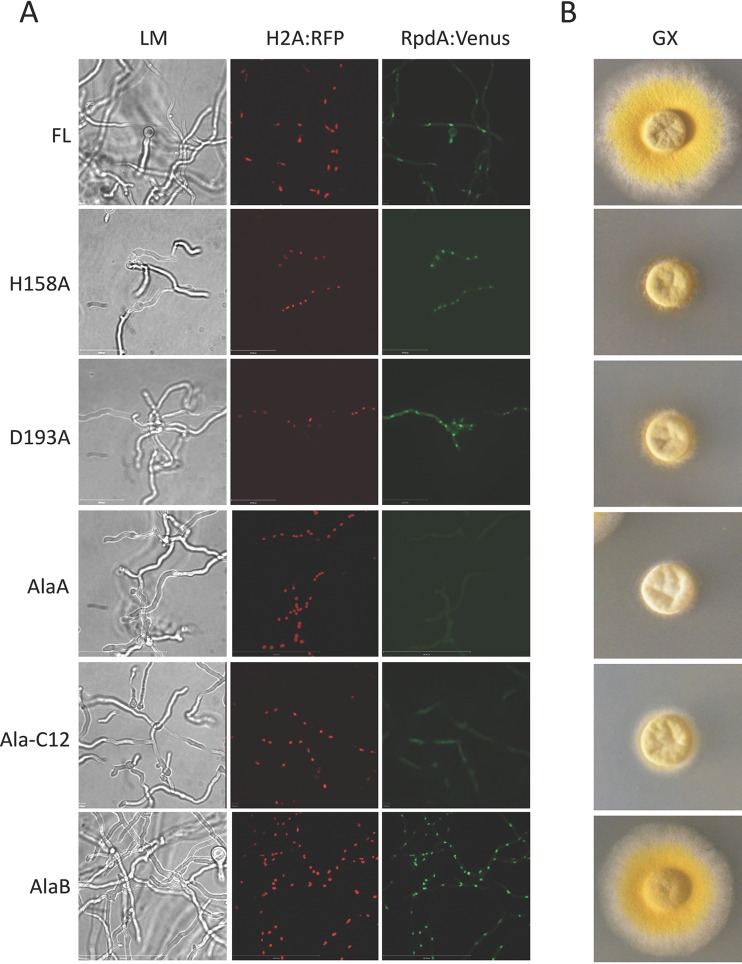
RpdA localization and phenotypic analysis of *Aspergillus* strains expressing Venus-tagged RpdA variants and mRFP-tagged histone H2A. Venus-tagged RpdA variants were expressed under the control of the *xylPp* promoter in strain TSG5 expressing endogenous RpdA under the control of *alcAp* and mRFP-tagged H2A under the control of the constitutive *gpdA* promoter. For microscopic analysis, strains were grown in eight-well plates (A), and for phenotypic analysis, they were grown on agar plates (B) under *xylPp* inductive conditions (GX). Hyphae were viewed under a light microscope (LM) at a magnification of ×630, and for subcellular localization of RpdA, they were examined by confocal laser scanning microscopy. Nuclei (mRFP-tagged H2A, H2A-mRFP) are red, and the distribution of expressed Venus-tagged RpdA variants (RpdA-Venus) is green.

Whereas class 2 HDACs shuttle between the nucleus and cytoplasm, RPD3 complexes act almost exclusively within the nucleus (44). In contrast to HDACs of higher eukaryotes, the mechanism of the nuclear transfer of HDACs in filamentous fungi is as yet unclear. To address the question of whether C12 is involved in cellular targeting of RpdA-type enzymes, strain TSG5 expressing histone H2A tagged with a red fluorescent protein (mRFP) driven by the *gpdA* promoter was transformed with expression cassettes for Venus-tagged wild-type (full-length) RpdA (RpdA-FL) or RpdA-ΔC12 under the control of *xylPp* (Fig. 2A). A codon-optimized sequence was used for expression of the yellow-green-fluorescent Venus protein in *A. nidulans*. Mutant strains grown under *alcAp*^−^/*xylPp*^+^ conditions were analyzed by confocal laser scanning microscopy. In contrast to tagged RpdA-FL (Fig. 7A) and the RpdA-ΔC6 control strain (see Fig. S5 in the supplemental material), both of which were enriched in the nucleus, RpdA-ΔC12 was randomly distributed throughout the hyphae (see Fig. S5 in the supplemental material). This result strongly suggests that C12 is essential for sufficient nuclear localization of RpdA. In order to pinpoint pivotal residues within this region, Venus-tagged RpdA fragments with C12 subdeletions, DelA to DelE, were expressed in the TSG5 recipient (Fig. 5B). With the exception of DelE (comprising six acidic residues), however, all of the RpdA variants were localized predominantly in the nucleus and able to compensate for full-length RpdA depletion (see Fig. S6 in the supplemental material). To prove that the deleterious effects of the deletion of C12 and DelE are indeed due to the loss of a negative charge, another three expression constructs, Ala-C12, AlaA, and AlaB, were generated. These constructs comprise the coding sequence of Venus-tagged RpdA with different alanine substitutions within the acidic patch (Fig. 5B). Strain TSG5 was transformed with the expression cassettes, and expression strains were again analyzed by fluorescence microscopy under *alcAp*^−^/*xylPp*^+^ conditions as described above. Interestingly, RpdA variants comprising at least five supplemented acidic residues (Ala-C12 and AlaA) led to reduced nuclear localization of the enzyme and to significant growth retardation of the corresponding expression strains (Fig. 7). On the other hand, strains expressing catalytic mutant RpdA proteins (H158A and D193A) or the RpdA C terminus alone (C-ter), including C12 (Fig. 5B), resembled the sick phenotype of the recipient, despite proper nuclear localization of the expression product (Fig. 7; see also Fig. S5 in the supplemental material). Altogether, these results indicate that a minimum of 5 out of 10 acidic residues within C18 is required for sufficient nuclear localization of RpdA; however, they also demonstrate that catalytic activity *per se* is not a prerequisite for nuclear accumulation of the HDAC.

### The acidic C-terminal region is required for full catalytic activity of RpdA.

**FIG 8:**
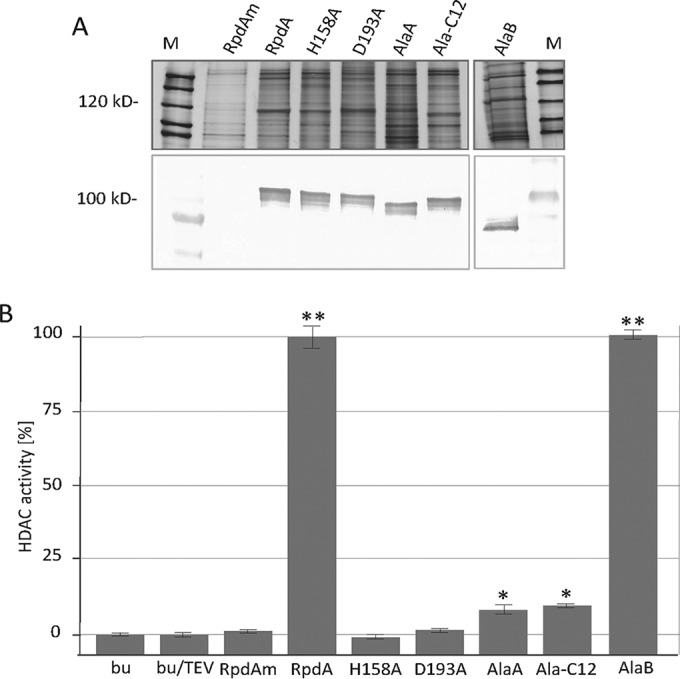
(A) HDAC activities of purified recombinant RpdA variants. IgG Sepharose-purified recombinant TAP-tagged RpdA variants were eluted by cleavage with TEV protease, and 10-µl aliquots of the eluates were subjected to SDS-PAGE, followed by silver staining of the proteins for quality control. For Western blotting, 5-µl volumes of the eluates were separated by SDS-PAGE and blotted onto a nitrocellulose membrane. RpdA expression products were detected with an anti-CBP antibody. An identically treated protein extract of a strain expressing Venus-tagged RpdA under the control of *xylPp* served as a mock-treated control (RpdAm). The molecular masses of relevant marker proteins (lanes M) are indicated. (B) Twenty-five-microliter volumes of the eluates were used for HDAC activity assays. The activity of wild-type RpdA was set to 100%, catalytic RpdA mutants (H158A, D193A) and RpdAm served as negative controls. Elution buffer (bu, with and without TEV protease) was used to determine background activity. The error bars represent standard deviations of three independent replicates. Asterisks indicate statistically significant differences from the buffer control (*, *P* < 0.001; **, *P* < 0.00001).

The fact that catalytic inactivation of RpdA led to growth defects similar to those caused by neutralization of the negatively charged C-terminal region prompted us to test for catalytic RpdA activity in Ala-C12 and AlaA strains. In particular, we were interested in whether neutralization of the acidic C-terminal region—although it is distant from the N-terminal catalytic domain—also affects the catalytic activity of RpdA. To address this question, RpdA variants Ala-C12 and AlaA were expressed as TAP-tagged proteins under the control of *xylPp* in recipient strain RIB214. After copurification of RpdA and associated complex partners under native conditions, eluted fractions were analyzed by SDS-gel electrophoresis and silver staining. The quantity and quality of expressed RpdA were assessed by immunoblotting with an anti-CBP antibody (Fig. 8A). As reported earlier, expressed RpdA migrates at a higher apparent molecular weight than predicted, most likely because of the specific properties of its acidic C-terminal part (24). Such changes in electrophoretic mobility during SDS-PAGE because of net negatively charged domains of proteins were recently examined in detail (45). Comparable amounts of purified recombinant RpdA variants were assayed for HDAC activity as described above. As a positive control, TAP-tagged wild-type RpdA was used; TAP-tagged catalytic mutant RpdA proteins (H158A and D193A) and Venus-tagged wild-type RpdA (mock control, RpdAm) served as negative controls. As expected, equal levels of HDAC activity were measured for the wild-type enzyme and AlaB, where only two of the negatively charged residues of C12 were substituted (Fig. 8B). No activity of the purified H158A or D193A enzyme was detectable. In contrast, both the Ala-C12 and AlaA RpdA variants showed catalytic HDAC activity above the background; however, activity was reduced to approximately 15% of that of the full-length enzyme. These results indicate that the negatively charged region of RpdA-type enzymes in filamentous fungi is not only essential for proper nuclear localization but also required for full catalytic activity.

## DISCUSSION

Formation of clinical multidrug resistance (CMR) of pathogenic microorganisms is not unique to prokaryotic pathogens but also impedes the treatment of fungal diseases (46). The limited arsenal of available antifungals and the generous use of similar substances against fungal crop plant diseases are mainly responsible for CMR in fungal pathogens. The development of early and more sensitive diagnostic tools and novel efficient antifungals is an opportunity to escape from this dilemma and will contribute to a continuative enhancement of antifungal therapies.

Similar to antibiotics used for the treatment of bacterial infections, the tolerability of antifungal therapy will be better when drugs do not interfere with important proteins or metabolic pathways of the patient. Amphotericin B, for a long time the first choice for the treatment of systemic aspergillosis, is frequently replaced with azole derivatives mainly because of its severe side effects. Increasing resistance to azoles, however, reduces their success and requires alternative therapies (47). Ideally, novel drugs will specifically target important enzymes or virulence factors of fungi without affecting their host.

The first evidence that HDACs play a decisive role as virulence factors was already shown in 2001 for the class 1 enzyme and HosA homolog HDC1 of the plant-pathogenic fungus *C. carbonum* (48). We were able to demonstrate that strains with *HDC1* deleted display significantly diminished pathogenicity on maize plants as a result of the reduced expression of extracellular depolymerases, which are required for the degradation of plant cell walls during infection (48). Subsequently, the impact of the corresponding enzyme in plant pathogenicity was confirmed in *Magnaporthe oryzae* and recently also in *Fusarium fujikuroi* (49, 50). Moreover, susceptibility testing of *Candida* and *Aspergillus* isolates has demonstrated that specific inhibition of HosA-type enzymes increased the sensitivity to azole derivatives of about 60% of the clinical isolates investigated (51).

In contrast to the deletion of RpdA, however, *hosA* null mutants are viable and even *hosA/hdaA* and *hosB/hdaA* double mutants of *A. nidulans* displayed no growth retardation comparable to that of RpdA-depleted strains (e.g., see references 26 and 31). The lethality of RpdA null mutants is raising the question of which RpdA targets are affected and responsible for this striking phenotype. In addition to their role as chromatin modifiers, classical HDACs were identified as regulators of posttranslational modifications of nonhistone proteins such as transcription factors and signal mediators. Among these substrates is heat shock protein 90 (Hsp90), a chaperon protein that was found to be acetylated in *A. fumigatus* predominantly at lysine 27 (K27). Interestingly, removal of this acetyl group is required for proper Hsp90 function (52). Since Hsp90 was found to be important for viability and resistance to certain antifungals of several fungal species (for reviews, see references 53 and 54), it might be that Hsp90 K27 is one of the crucial targets of RpdA. However, further work is required to prove this assumption and identify further functions that make this enzyme indispensable for filamentous fungi.

Irrespective of its specific biological function, the impact of RpdA for pathogenic species such as *A. fumigatus* and the finding that RpdA inhibition by TSA significantly delays growth and germination turn HDACIs into a promising class of drugs for the treatment of fungal infections.

Because of their increasing importance as cytostatic agents that inhibit the proliferation of tumor cells, several potent HDACIs are currently in clinical trials or already FDA approved as therapy for specific types of cancer (55, 56). Experience arising from these trials will facilitate the use of HDACIs for other clinical applications such as the treatment of mycosis. In this context, it is important to note that most of these substances act as pan-inhibitors that target the N-terminal catalytic domain that is highly conserved in the classical HDACs of all eukaryotes (41). HDACI for antifungal therapies, in contrast to anticancer use, should primarily aim to target fungal HDACs, preferably RpdA, since this enzyme is essential. To achieve this, profound knowledge about differences between the structures, complex partners, and biological functions of fungal HDACs and their orthologs in higher eukaryotes is required.

Our complementation studies clearly demonstrate that the C terminus of RpdA comprises a fungus-specific charged region that, when mutated, causes biological inactivity and an atrophic phenotype with drastic restriction of the radial growth of mutant strains. Our data strongly suggest that neutralization of acidic residues within this region leads to misfolding of RpdA and consequently to (i) disturbed nuclear localization and (ii) significantly decreased catalytic activity. Vice versa, strains with point mutations in the catalytically active N-terminal domain display an identical lethal phenotype, although the (inactive) enzyme is properly located in the nucleus. This is important with respect to recent findings on RPD3 complexes in *S. cerevisiae*. A study revealed that two of the three RPD3 complexes identified in yeast possess a previously unrecognized ability to promote nucleosome assembly in the sense of a histone chaperone. Hence, RPD3 contributes to transcriptional repression via a nucleosome-stabilizing function independently of its catalytic activity (38). The results of our study indicate that both appropriate nuclear enrichment and catalytic activity of RpdA are required for sufficient growth and development of filamentous fungi.

In contrast to a catalytic domain that is widely conserved in all eukaryotic enzymes, nuclear localization of RpdA depends on a charged motif that is missing from orthologous enzymes of yeasts and higher eukaryotes and the same region is also indispensable for the full HDAC activity of the enzyme. Consequently, even RpdA-type proteins of quite distantly related filamentous fungi, but not those of higher eukaryotes or yeasts, were able to restore the phenotype of RpdA-depleted strains.

The absence of the fungus-specific C-terminal motif from RPD3-type enzymes of higher eukaryotes, however, raises the question of how nuclear localization is achieved in those organisms. In murine HDAC1, a C-terminal lysine-rich nuclear localization signal (NLS) has been found to be sufficient for nuclear import (57). Alternatively, HDAC1 can be transported into the nucleus via another HDAC1 molecule that is bound by an N-terminal histone association domain (HAD) and, in contrast to fungi, mammalian HDAC1 is commonly associated with another class 1 enzyme, HDAC2, in the same complex. The latter also possesses a basic stretch of amino acids considered to be an NLS. Thus, murine HDAC1 can be imported into the nucleus in the absence of its internal NLS via a piggyback mechanism and only the removal of both the HAD and the NLS retains the enzyme in the cytoplasm.

In the maternal class 1 HDAC HDACm of *Xenopus* oocytes, a bipartite C-terminal sequence that plays a critical role in nuclear uptake has been described (58). Mutation of a single tyrosine (T445) in the center of this bipartite NLS leads to a 5-fold reduction in the rate of translocation of the enzyme into the germinal vesicle. Interestingly, T445 was identified as one of several target sites of cytoplasmic protein kinase 2 (CK2) and phosphorylation of this residue seems to be a crucial step for nuclear import. Moreover, phosphorylation of another two proximal sites, serine 421 (S421) and serine 423 (S423), affects the release of HDACm from the import receptor and is required for catalytic activity of the enzyme. Phosphorylation of these two serine residues was also found to be important for the full enzymatic activity and proper complex formation of human HDAC1 (59).

Interestingly, these data contradict those of fungi. Whereas the short C terminus of *S. cerevisiae* RPD3 contains neither an NLS nor phosphorylation sites comparable to those described in higher eukaryotes, enzymes of filamentous fungi do comprise at least one of these serine residues and also one putative NLS (or even two) (see Fig. S8 in the supplemental material).

An RpdA fragment truncated N terminally of those serines, however, was able to restore the wild-type phenotype of RpdA-depleted *A. nidulans* mutants (31). Consequently, serine phosphorylation does not seem to be essential for the biological functionality of RpdA-type enzymes in fungi. Moreover, a bipartite C-terminal NLS found to be crucial for the nuclear transport of HDACs of higher eukaryotes is missing from fungal enzymes. Nevertheless, one or even two putative NLSs were predicted in proximity to the acidic region or in the N terminus of some RpdA-type proteins by NLS mapper software, but the number and localization depend on the fungal species investigated (see Fig. S8 in the supplemental material). RpdA proteins of aspergilli, for example, comprise two putative NLSs; however, deletion or mutation did not lead to significant nuclear depletion of RpdA in *A. nidulans* (data not shown). Instead, the fungus-specific acidic stretch in *A. nidulans* is required for both full catalytic activity and proper nuclear localization of the enzyme. Although the detailed mechanism remains to be elucidated, at least two possibilities for how nuclear entry of RpdA might occur are conceivable. Either RpdA binds to complex partners responsible for its nuclear targeting via a piggyback mechanism, as described for other fungal proteins (e.g., see reference 60), or it is directly transferred into the nucleus by a still unidentified NLS. In addition, it might also be conceivable that increased nuclear export is responsible for the nuclear depletion of RpdA in the corresponding mutant strains (61). In any case, the acidic region might be crucial for appropriate folding that is involved in the binding of karyopherins or other carrier proteins.

The fact that recombinant RpdA-type proteins of *Cochliobolus*, *Penicillium*, and *Neurospora* are sufficiently enriched in the nucleus (see Fig. S7 in the supplemental material) and are able to complement RpdA minus mutants of *A. nidulans* (Fig. 6) suggests that the molecular mechanisms of nuclear trafficking and the biological functions of RpdA-type proteins are very similar in filamentous fungi.

Its crucial role for filamentous fungi makes RpdA a promising target for HDACIs with antifungal activity. As shown for SAHA, however, not all medically established HDACIs might be suitable to inhibit RpdA activity or fungal growth effectively. Ongoing analyses with clinically approved and novel HDACIs will elucidate their efficacy against affinity-purified RpdA activity *in vitro*, and only the most effective substances will be suitable candidates for further testing of their potential as antifungal substances *in vivo*. The successful identification of fungus-specific HDACIs as new agents for the prevention or therapy of invasive fungal infections will definitely be a desirable goal of upcoming research projects.

## MATERIALS AND METHODS

### Fungal strains and growth media.

The fungal strains used in this study are listed in Table S1 in the supplemental material. *A. nidulans* strains generated for expression of (mutated) RPD3-type HDACs were derived from A768 and A89, both provided by the Fungal Genetics Stock Center (Kansas City, KS). The *A. fumigatus* strains used for TSA inhibition experiments and for heterokaryon rescue are wild-type derivatives. Unless otherwise noted, strains were grown at 37°C in *Aspergillus* MM, *Penicillium* MM, Vogel’s medium, or RPMI 1640 medium containing appropriate supplementation as described previously (62).

### TSA inhibitor assay in liquid cultures.

For TSA inhibitor testing, RPMI 1640 medium was inoculated with *A. fumigatus* A1163 (1 × 10^5^/ml) in 24-well plates (0.9 ml/well) and incubated at 37°C. Different concentrations of TSA (Sigma T1952, 5 mM stock solution in DMSO) were added immediately or 10 h after inoculation. Nontreated cultures were supplemented with corresponding volumes of DMSO. Germination and growth were evaluated at a magnification of ×200 by using a Leica DMIL LED inverted microscope and documented with AxioVision 4.8 software (Zeiss).

### HDACI assay on solid medium.

Strains were grown in 12-well plates containing 1.5 ml of solid medium per well point inoculated with 2 µl of a conidial suspension (5 × 10^5^/ml) and incubated overnight at different temperatures to allow for germination. Colonies were then overlaid with 100 µl of liquid medium containing either the inhibitor at a concentration calculated for 1.6 ml or the respective volume of DMSO as a control. Plates were then incubated at 37°C or 25°C and evaluated after different times. In addition to TSA (see above), the inhibitors vorinostat (SAHA, Selleckchem SD1047, 150 mM stock solution in DMSO) and apicidin (Sigma A8851, 1.5/3.75 mM stock solution in DMSO) were used.

### Generation of expression constructs.

For cloning, amplification, digestion, and propagation of DNA fragments and vectors, standard molecular techniques were used. Generation of RpdA expression constructs was achieved by fusion of the heterologous xylanase promoter (*xylPp*) of *P. chrysogenum* to the coding sequence of the RPD3-type HDAC to be expressed (31). To this end, *xylPp* was amplified by PCR and fused to the amplified coding sequence of the HDAC by overlap extension PCR as described previously (63) or by In-Fusion HD Cloning (Clontech Laboratories, Inc.) in accordance with the manufacturer’s instructions. For deletion or alanine substitution of residues within the RpdA C terminus, the *xylPp*-*rpdA* expression vector was digested with ClaI and HpaI and the 3′ fragment of *rpdA* (comprising the motif to be mutated) was gel purified and inserted into a vector. This vector was used as the template for a PCR with primers annealing directly up- and downstream of the site to be deleted. For the substitution of acidic residues, alanine codons were added to the 5′ side of both primers. Subsequently, the amplified *rpdA/*vector fragment was religated at the modified site and used for transformation of *Escherichia coli*. After preparation of the plasmid, the mutated *rpdA* fragment was sequenced and introduced into the *xylPp*-*rpdA* expression vector again by using the ClaI and HpaI sites. For selection of fungal transformants, the expression constructs contained an *argB* marker gene encoding either a functional (64) or a nonfunctional (65) ArgB protein for random or targeted integration at the *argB* locus, respectively.

### Heterokaryon rescue experiment.

The heterokaryon rescue experiment was performed as described previously (32), with minor modifications. For generation of the mutant allele, the split-marker technique was used (66). *A. fumigatus* A1280 (ATCC 46645 Δ*akuA*) was cotransformed with two DNA fragments, each containing 0.5-kb overlapping but incomplete fragments of the pyrithiamine resistance-conferring *ptrA* allele from *Aspergillus oryzae* (67) ligated to 1.2- and 1.5-kb *A. fumigatus* rpdA 5′- and 3′-flanking regions, respectively. Amplification of the fragments was performed with the PfuX7 polymerase (68) with plasmid pIB25 harboring the *A. fumigatus* rpdA flanking regions fused to the *ptrA* gene as the template. Originally, the *ptrA* marker was released from plasmid pSK275 by digestion with NdeI and PstI. In the resulting mutant allele, residues −57 to 2269 relative to the *rpdA* translation start are deleted. Transformation of *A. fumigatus* was done as described elsewhere (69). Pyrithiamine (0.1 µg/ml) was used for selection of transformants and confirmation of heterokaryon rescue.

### Transformation and screening of the transformants.

Transformation of *A. nidulans* strains RIB211, RIB214, and TSG5 was done with 5 to 10 µg of expression constructs as described previously (69). Protoplasts were recovered on medium with 3% lactose as a carbon source and 10 mM l-threonine to ensure the expression of functional RpdA driven by the *alcA* promoter. Genomic DNA was prepared from colonies deriving from mycelia of homokaryotic spores of the transformants and used for PCR screening. From PCR-positive strains, genomic (single) integration was confirmed by Southern blot analysis. Preparation of genomic DNA and Southern blot analysis were performed as described earlier (22). The ability of *xylPp*-regulated RpdA variants or heterologous RPD3-type enzymes to compensate for depleted RpdA under the control of *alcAp* was tested on medium with 1% glucose and 1% xylose as previously described (31). Expression of the recombinant proteins was verified by Northern and Western blot analyses, respectively.

### Northern and Western blot analyses.

The transcription and translation of recombinant HDACs were analyzed under *xylPp* inductive and repressive conditions. RNA preparation, blotting, and hybridization were done as described previously (22). Hybridized, digoxigenin-dUTP-labeled DNA probes were detected with alkaline phosphatase-conjugated anti-digoxigenin Fab fragments (Roche) and developed with CSPD chemiluminescent substrate (Roche) according to the manufacturer’s instructions. Signals were visualized by exposure to X-ray film or with the Fusion-SL 3500 WL imaging system (Vilber Lourmat).

Total protein extracts were prepared by grinding 50 to 100 mg of lyophilized mycelia with a tungsten carbide ball in a mixer mill (Retsch MM 400), followed by extraction with 250 to 500 µl of buffer B250 (for composition, see below). Western blotting and detection were performed as described in reference 24. Proteins were detected with antibodies directed against the RpdA C terminus (24), anti-CBP (Millipore 07-482, 1:1,333), or anti-human HDAC1 (Zymed 34-8300, 1:1,000).

### Sexual crosses of *A. nidulans*.

To generate recipient strains RIB211 and RIB214, corresponding parent strains were crossed as listed in Table S1 in the supplemental material. Crosses were done as described in reference 70.

### Confocal laser scanning microscopy.

Conidia (1 × 10^4^) were incubated in 0.2 ml of glucose-containing MM with 0.5 to 1% xylose in eight-well chambered coverglasses (Nunc Lab-Tek, Thermo Scientific) at 30°C without shaking overnight. Mycelia were examined with a spinning-disc confocal microscopic system (Ultra VIEW VoX; PerkinElmer, Waltham, MA) that was connected to a Zeiss AxioObserver Z1 inverted microscope (Zeiss). Images were acquired with Volocity software (PerkinElmer) with a 63× oil immersion objective with a 1.42 numerical aperture. The laser wavelengths used for excitation of Venus and mRFP were 488 and 561 nm, respectively.

### Purification of RpdA activity and HDAC assay.

Affinity purification was performed as described in reference 71. Eight 1,000-ml Erlenmeyer flasks each containing 200 ml of GX-MM were inoculated with 5 × 10^6^ conidia per ml. Mycelia were grown at 37°C at 220 rpm for 12 h and subsequently harvested by filtration, washed with harvest solution (0.96% NaCl, 1 mM phenylmethylsulfonyl fluoride [PMSF]), frozen in liquid nitrogen, and ground with a mortar and pestle. All of the following steps were performed at 4°C. Ground mycelia were extracted in buffer B250 (250 mM NaCl, 100 mM Tris-HCl [pH 7.5], 10% glycerol, 0.5 mM EDTA, 0.1 mM PMSF, 5 mM 2-mercaptoethanol, 1× Roche Complete protease inhibitors). After centrifugation at 30,000 × *g* for 30 min, clarified crude lysate was incubated with IgG Sepharose 6 Fast Flow resin (GE Healthcare) for 4 h at 4°C on a rotor (ELMI RM-2 S Intelli Mixer) and afterward transferred into a 10-ml chromatography column (Bio-Rad Poly-Prep). The IgG Sepharose was washed twice with 10 ml of buffer W250 (250 mM NaCl, 40 mM Tris-HCl [pH 7.5], 0.1% Triton X-100, 5 mM 2-mercaptoethanol), once with 10 ml of buffer WB150 (150 mM NaCl, 40 mM Tris-HCl [pH 7.5], 0.1% Triton X-100, 5 mM 2-mercaptoethanol), and subsequently with 10 ml of TCB (150 mM NaCl, 40 mM Tris-HCl [pH 7.5], 0.1% Triton X-100, 1 mM DTT, 0.5 mM EDTA [pH 8], 1× Roche Complete protease inhibitors). Elution from the IgG beads occurred by tobacco etch virus (TEV) cleavage by incubating the IgG Sepharose overnight in 1 ml of TCB containing 50 µl of TEV protease (1 mg/ml) at 4°C on a rotor. Aliquots of the elution were directly used for HDAC assays or frozen in liquid nitrogen for storage at −80°C. To assay for HDAC activity, 25 µl of the eluate was mixed with 10 µl of total [^3^H]acetate-prelabeled chicken reticulocyte histones (4 mg/ml) as the substrate in a total volume of 60 µl (24). After incubation for 1 h at 25°C, the reaction was stopped by the addition of 50 µl of 1 M HCL–0.4 M acetate and 0.8 ml of ethyl acetate. Released acetyl groups were extracted with 800 µl of ethyl acetate. After centrifugation at 10,000 × *g* for 10 min, the radioactivity of an aliquot of 600 µl of the upper phase was counted in 3 ml of liquid scintillation cocktail (Rotiszint eco plus) in a Hitachi Aloka AccuFlex LSC-8000 scintillation counter.

### Statistical analysis.

Analysis of the statistical significance of differences in catalytic activity between purified RpdA variants and the reference (buffer) was done by Student *t* test in R (version 3.4.2) and RStudio version 0.99.491 (https://www.r-project.org).

## SUPPLEMENTAL MATERIAL

Figure S1  Classical HDACs in aspergilli. Classical HDACs are divided into class 1 (RPD3-type) and 2 (HDA1-type) enzymes that show broad sequence similarity to each other mainly because of a highly conserved N-terminal catalytic domain. Aspergilli and many other filamentous fungi have two class 1-type HDACs (RpdA and HosA—homologous to yeast RPD3 and HOS2) and two class 2-type HDACs (HdaA and HosB—homologous to yeast HDA1 and HOS2), respectively. A homologous enzyme of yeast HOS3 in missing from filamentous fungi. RpdA, the subject of this study, is shown in bold. Download Figure S1, PDF file, 0.1 MB

Figure S2  Alignments of N-terminal regions of RPD3-type HDACs. The N-terminal extension (N18) and the C-terminal motif (C18, represented in more detail in Fig. 5B) specific for *A. nidulans* and other filamentous fungi are shown as white boxes in the schematic representation of *A. nidulans* RpdA (center). The highly conserved catalytic part of HDACs comprising several amino acids essential for the catalytic activity of HDACs of all eukaryotes is depicted in gray. The stretch deleted in ΔN18 RpdA is boxed in red in the alignment with human HDAC1 (A). Histidine 158 (H158) and asparagine 193 (D193), which were modified to alanine in the catalytically inactivated *A. nidulans* mutants, are boxed in red in the alignment (B). RPD3-type sequences from *A. nidulans* (*Ani*), *A. terreus* (*Ate*), *A. fumigatus* (*Afu*), *N. crassa* (*Ncr*), *P. chrysogenum* (*Pch*), *C. carbonum* (*Cca*), *X. laevis* (*Xla*), *H. sapiens* (*Hsa*), and *Z. mays* (*Zma*) are shown. The intensity of the gray coloring depends on the grade of identity of the aligned residues. Download Figure S2, PDF file, 0.1 MB

Figure S3  TSA efficacy and stability testing with affinity-purified RpdA activity. The efficacy of RpdA inhibition was tested with 25 µl of affinity-purified wild-type RpdA and 0, 10, 50, and 500 nM concentrations of the inhibitor diluted in RPMI. The stability of TSA under culture conditions was examined with 50 nM TSA from an *A. fumigatus* culture (RPMI with 50 µM TSA; see Fig. 3B) after 0, 5, and 24 h of growth. Download Figure S3, PDF file, 0.02 MB

Figure S4  Efficacy of the HDACIs SAHA (vorinostat) and apicidin in comparison to TSA (A) and effect on the growth and conidiation of *A. fumigatus* (B). The efficacy of RpdA inhibition was tested with 25 µl of purified wild-type RpdA and the inhibitor at 0, 50, and 500 nM (A). Conidia of *A. fumigatus* (1 × 10^3^) were dotted onto the middle of each agar well, and strains were grown overnight to allow germination. Subsequently, colonies were overlaid with 100 µl of liquid medium containing an appropriate concentration of the inhibitor. A corresponding concentration of DMSO was used as a negative control. After incubation for 24 and 44 h at 37°C, colony size and conidiation of the mycelium were assessed (B). Download Figure S4, PDF file, 0.1 MB

Figure S5  Localization of the expressed RpdA C terminus and of RpdA with deletion of charged C-terminal regions C12 and C6, respectively. Venus-tagged RpdA variants were expressed under the control of *xylPp* in strain TSG5 comprising mRFP-tagged H2A under the control of the *gpdA* promoter. For microscopic analysis, strains were grown on coverglasses in eight-well plates under *xylPp* inductive conditions. Hyphae were viewed under a light microscope (LM) and also, for subcellular localization of the expression products, examined by confocal laser scanning microscopy at a magnification of ×630. Nuclei (H2A-mRFP) are red, and the distribution of expressed Venus-tagged RpdA variants del-C6 and del-C12 and the RpdA C terminus (C-Ter) is shown in green (RpdA-Venus). Download Figure S5, PDF file, 0.1 MB

Figure S6  Localization of RpdA variants with different deletions within fungus-specific, acidic region C12. Venus-tagged RpdA variants were expressed under the control of *xylPp* in strain TSG5 comprising mRFP-tagged H2A under the control of the *gpdA* promoter. For microscopic analysis, strains were grown on coverglasses in eight-well plates under *xylPp* inductive conditions. Hyphae were viewed under a light microscope (LM) and also, for subcellular localization of the RpdA variants, examined by confocal laser scanning or epifluorescence microscopy (DelE) at a magnification of ×630. Nuclei (H2A-mRFP) are red, and the distribution of expressed Venus-tagged RpdA variants (RpdA-Venus) is shown in green. Download Figure S6, PDF file, 0.1 MB

Figure S7  Localization of RPD3-type HDACs expressed in *A. nidulans*. Venus-tagged heterologous HDACs of *A. nidulans* (*Ani*, positive control), *P. chrysogenum* (*Pch*), *N. crassa* (*Ncr*), and *Homo sapiens* (*Hsa*) were expressed under the control of *xylPp* in strain TSG5 (*Ani*, *Pch*, *Ncr*) or RIB214 (*Hsa*). For microscopic analysis, strains were grown on coverglasses in eight-well plates under *xylPp* inductive conditions. Hyphae were viewed under a light microscope (LM) and also, for subcellular localization of the RpdA homologous enzymes, examined by confocal laser scanning or epifluorescence microscopy (*Hsa*) at a magnification of ×630. Nuclei (H2A-mRFP) are red, and the distribution of expressed Venus-tagged RpdA variants (RpdA-Venus) is shown in green. nd, not determined (no H2A-mRFP strain generated). Download Figure S7, PDF file, 0.1 MB

Figure S8  Predicted nuclear localization signals of fungal RPD3-type HDACs. A schematic representation of *S. cerevisiae* (*Sce*) RPD3, the RpdA-type enzymes of *A. nidulans* (*Ani*) and its C terminus, (C-ter), *A. fumigatus* (*Afu*), *A. terreus* (*Ate*), *N. crassa* (*Ncr*), *P. chrysogenum* (*Pch*), and *C. carbonum* (*Cca*) is shown. Importin α-dependent NLSs and bipartite NLS (bNLS) predicted by cNLS mapper software (72) are depicted in yellow. Possible phosphorylation sites (S) according to the corresponding serine residues of mammalian HDAC1 are indicated. The highly conserved region comprising amino acid residues essential for catalytic activity is shown in gray, and the acidic C-terminal stretch conserved in filamentous fungi (C18) is represented by a white box. Distances between motifs and sites are not drawn to scale. Download Figure S8, PDF file, 0.02 MB

Table S1  Genotypes of strains used for the expression of RpdA variants (A) and heterologous RPD3-type HDACs (B) or in TSA inhibition and heterokaryon rescue experiments (C). The lab name and the pseudonym used in this report are specified. The genetic characteristic of strains are indicated as *rpdA*p::*pyrG*-*alcAp*-*rpdA* [*alcA*(*p*)-*rpdA*], which indicates replacement of the endogenous *rpdA* promoter (*rpdAp*) with the alcohol dehydrogenase promoter (*alcAp*). In this case, *pyrG* was used as an auxotrophic marker for selection of transformants. For expression strains, the corresponding expression plasmids and the selection marker used are shown. The gene in the construct to be expressed under the control of the xylanase promoter (*xylPp*) of *P. chrysogenum* is indicated in the fourth column. Strains H4, A18, A89, and RIB211 were used for sexual crosses. Experiments were done with at least three independent transformants of each genotype shown. Table S1, PDF file, 0.1 MB
