# Supplementary figures and images for: A Class 1 Histone Deacetylase with Potential as an Antifungal Target
Source: mBio. 2016 Nov 1;7(6):e00831-16. doi: 10.1128/mBio.00831-16 (PMC5090035; doi:10.1128/mBio.00831-16)

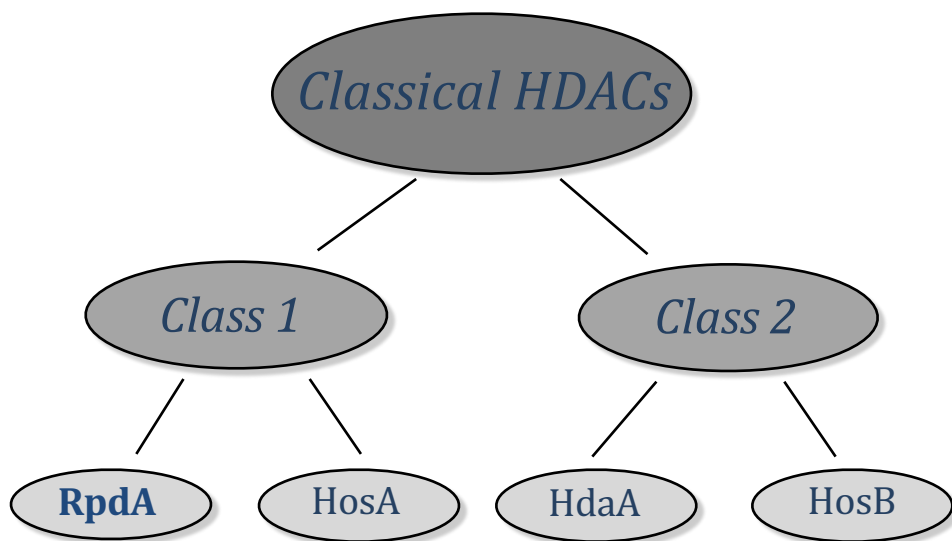

**Figure S1** – Classical histone deacetylases (HDACs) in *Aspergilli*.

Supplement: Figure S1 — Classical HDACs in aspergilli. Classical HDACs are divided into class 1 (RPD3-type) and 2 (HDA1-type) enzymes that show broad sequence similarity to each other mainly because of a highly conserved N-terminal catalytic domain. Aspergilli and many other filamentous fungi have two class 1-type HDACs (RpdA and HosA—homologous to yeast RPD3 and HOS2) and two class 2-type HDACs (HdaA and HosB—homologous to yeast HDA1 and HOS2), respectively. A homologous enzyme of yeast HOS3 in missing from filamentous fungi. RpdA, the subject of this study, is shown in bold. Download [file mbo005163048sf1.pdf]

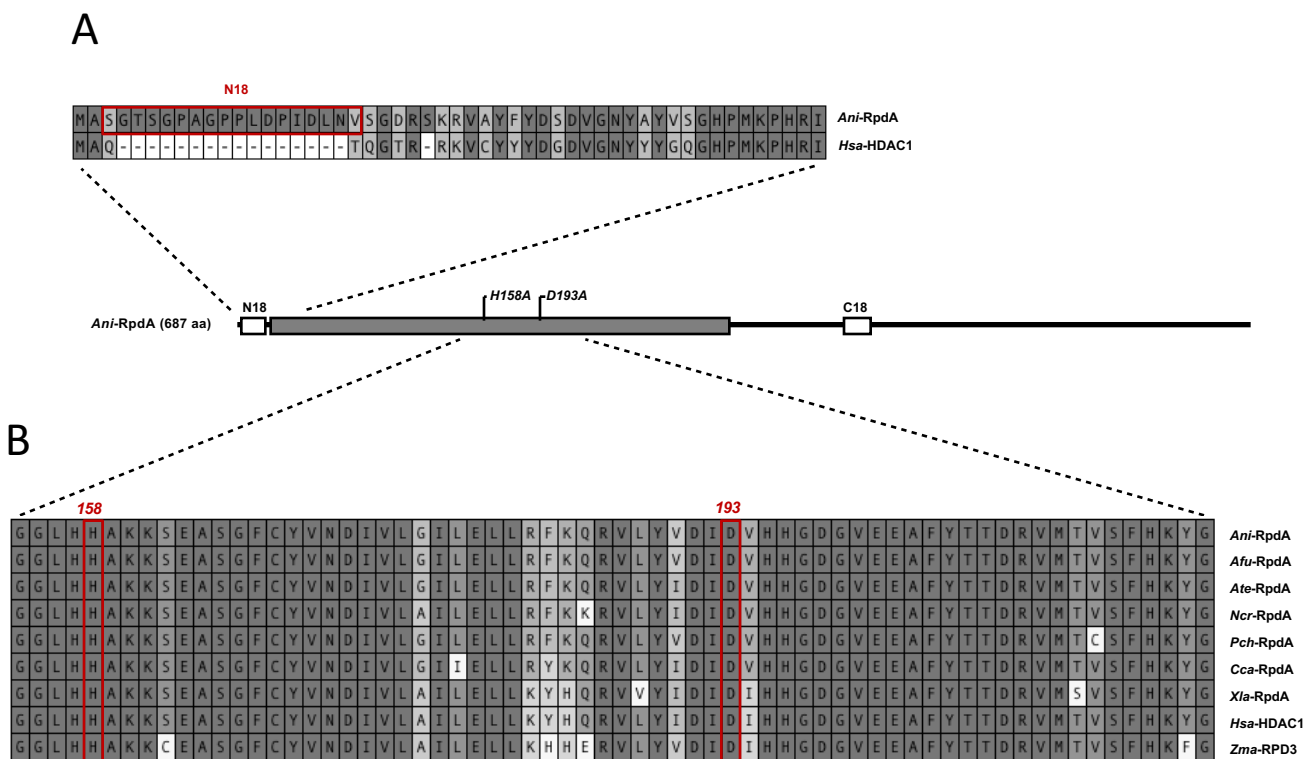

**Figure S2** – Alignments of N-terminal regions of RPD3-type HDACs.

Supplement: Figure S2 — Alignments of N-terminal regions of RPD3-type HDACs. The N-terminal extension (N18) and the C-terminal motif (C18, represented in more detail in Fig. 5B) specific for A. nidulans and other filamentous fungi are shown as white boxes in the schematic representation of A. nidulans RpdA (center). The highly conserved catalytic part of HDACs comprising several amino acids essential for the catalytic activity of HDACs of all eukaryotes is depicted in gray. The stretch deleted in ΔN18 RpdA is boxed in red in the alignment with human HDAC1 (A). Histidine 158 (H158) and asparagine 193 (D193), which were modified to alanine in the catalytically inactivated A. nidulans mutants, are boxed in red in the alignment (B). RPD3-type sequences from A. nidulans (Ani), A. terreus (Ate), A. fumigatus (Afu), N. crassa (Ncr), P. chrysogenum (Pch), C. carbonum (Cca), X. laevis (Xla), H. sapiens (Hsa), and Z. mays (Zma) are shown. The intensity of the gray coloring depends on the grade of identity of the aligned residues. Download [file mbo005163048sf2.pdf]

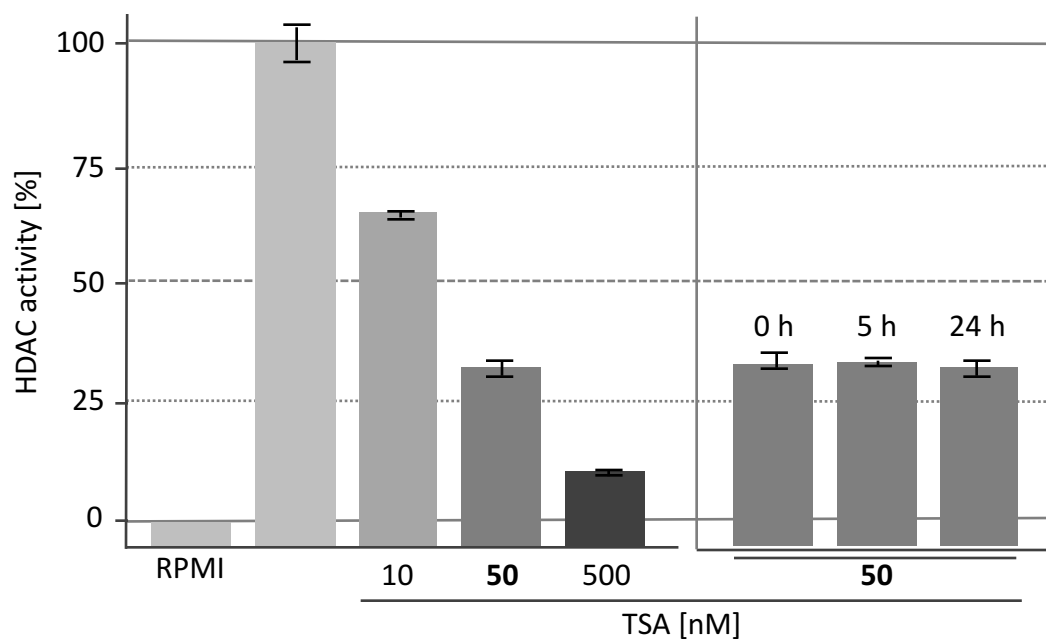

**Figure S3** – Efficacy and stability testing of TSA with affinity-purified RpdA activity.

Supplement: Figure S3 — TSA efficacy and stability testing with affinity-purified RpdA activity. The efficacy of RpdA inhibition was tested with 25 µl of affinity-purified wild-type RpdA and 0, 10, 50, and 500 nM concentrations of the inhibitor diluted in RPMI. The stability of TSA under culture conditions was examined with 50 nM TSA from an A. fumigatus culture (RPMI with 50 µM TSA; see Fig. 3B) after 0, 5, and 24 h of growth. Download [file mbo005163048sf3.pdf]

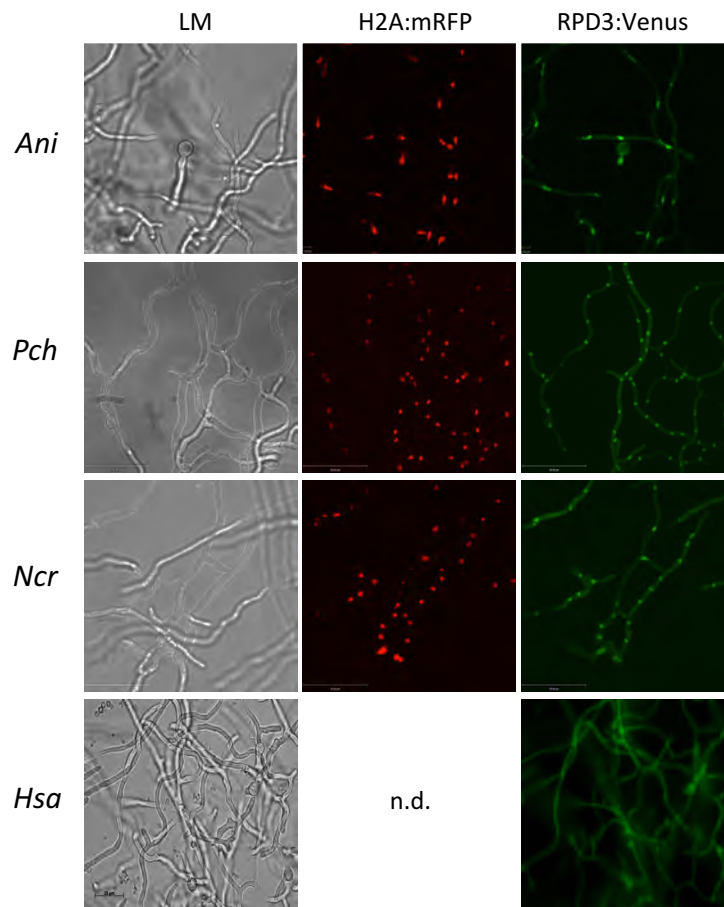

**Figure S7** – Localization of heterologous RPD3-type HDACs expressed in *Aspergillus nidulans*.

Supplement: Figure S7 — Localization of RPD3-type HDACs expressed in A. nidulans. Venus-tagged heterologous HDACs of A. nidulans (Ani, positive control), P. chrysogenum (Pch), N. crassa (Ncr), and Homo sapiens (Hsa) were expressed under the control of xylPp in strain TSG5 (Ani, Pch, Ncr) or RIB214 (Hsa). For microscopic analysis, strains were grown on coverglasses in eight-well plates under xylPp inductive conditions. Hyphae were viewed under a light microscope (LM) and also, for subcellular localization of the RpdA homologous enzymes, examined by confocal laser scanning or epifluorescence microscopy (Hsa) at a magnification of ×630. Nuclei (H2A-mRFP) are red, and the distribution of expressed Venus-tagged RpdA variants (RpdA-Venus) is shown in green. nd, not determined (no H2A-mRFP strain generated). Download [file mbo005163048sf7.pdf]

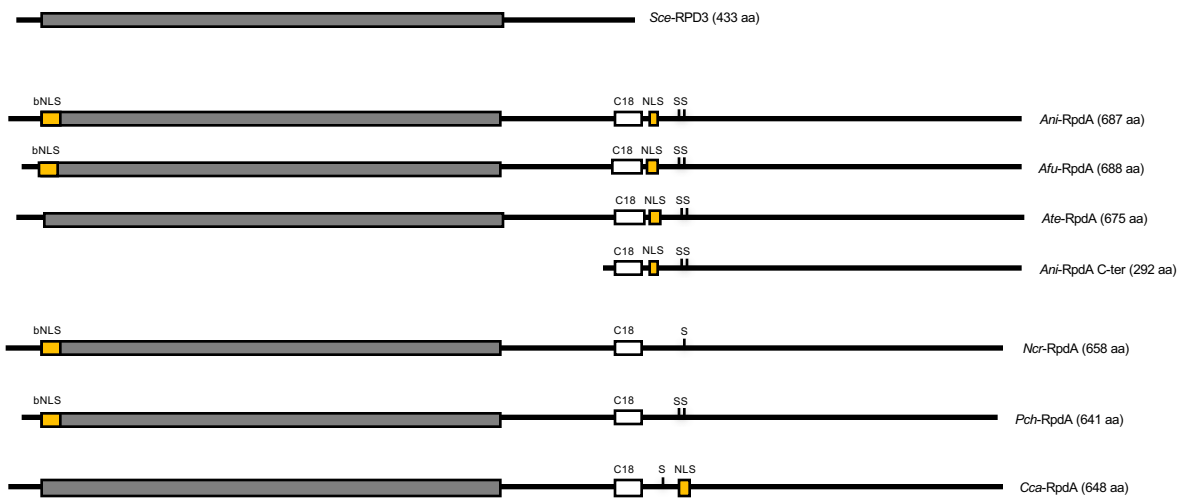

**Figure S8** – Predicted nuclear localization signals of fungal RPD3-type HDACs.

Supplement: Figure S8 — Predicted nuclear localization signals of fungal RPD3-type HDACs. A schematic representation of S. cerevisiae (Sce) RPD3, the RpdA-type enzymes of A. nidulans (Ani) and its C terminus, (C-ter), A. fumigatus (Afu), A. terreus (Ate), N. crassa (Ncr), P. chrysogenum (Pch), and C. carbonum (Cca) is shown. Importin α-dependent NLSs and bipartite NLS (bNLS) predicted by cNLS mapper software (72) are depicted in yellow. Possible phosphorylation sites (S) according to the corresponding serine residues of mammalian HDAC1 are indicated. The highly conserved region comprising amino acid residues essential for catalytic activity is shown in gray, and the acidic C-terminal stretch conserved in filamentous fungi (C18) is represented by a white box. Distances between motifs and sites are not drawn to scale. Download [file mbo005163048sf8.pdf]
