# Supplementary material for: A Class 1 Histone Deacetylase with Potential as an Antifungal Target
Source: mBio. 2016 Nov 1;7(6):e00831-16. doi: 10.1128/mBio.00831-16 (PMC5090035; doi:10.1128/mBio.00831-16)
Supplement: Figure S4 — Efficacy of the HDACIs SAHA (vorinostat) and apicidin in comparison to TSA (A) and effect on the growth and conidiation of A. fumigatus (B). The efficacy of RpdA inhibition was tested with 25 µl of purified wild-type RpdA and the inhibitor at 0, 50, and 500 nM (A). Conidia of A. fumigatus (1 × 10^3^) were dotted onto the middle of each agar well, and strains were grown overnight to allow germination. Subsequently, colonies were overlaid with 100 µl of liquid medium containing an appropriate concentration of the inhibitor. A corresponding concentration of DMSO was used as a negative control. After incubation for 24 and 44 h at 37°C, colony size and conidiation of the mycelium were assessed (B). Download [file mbo005163048sf4.pdf]

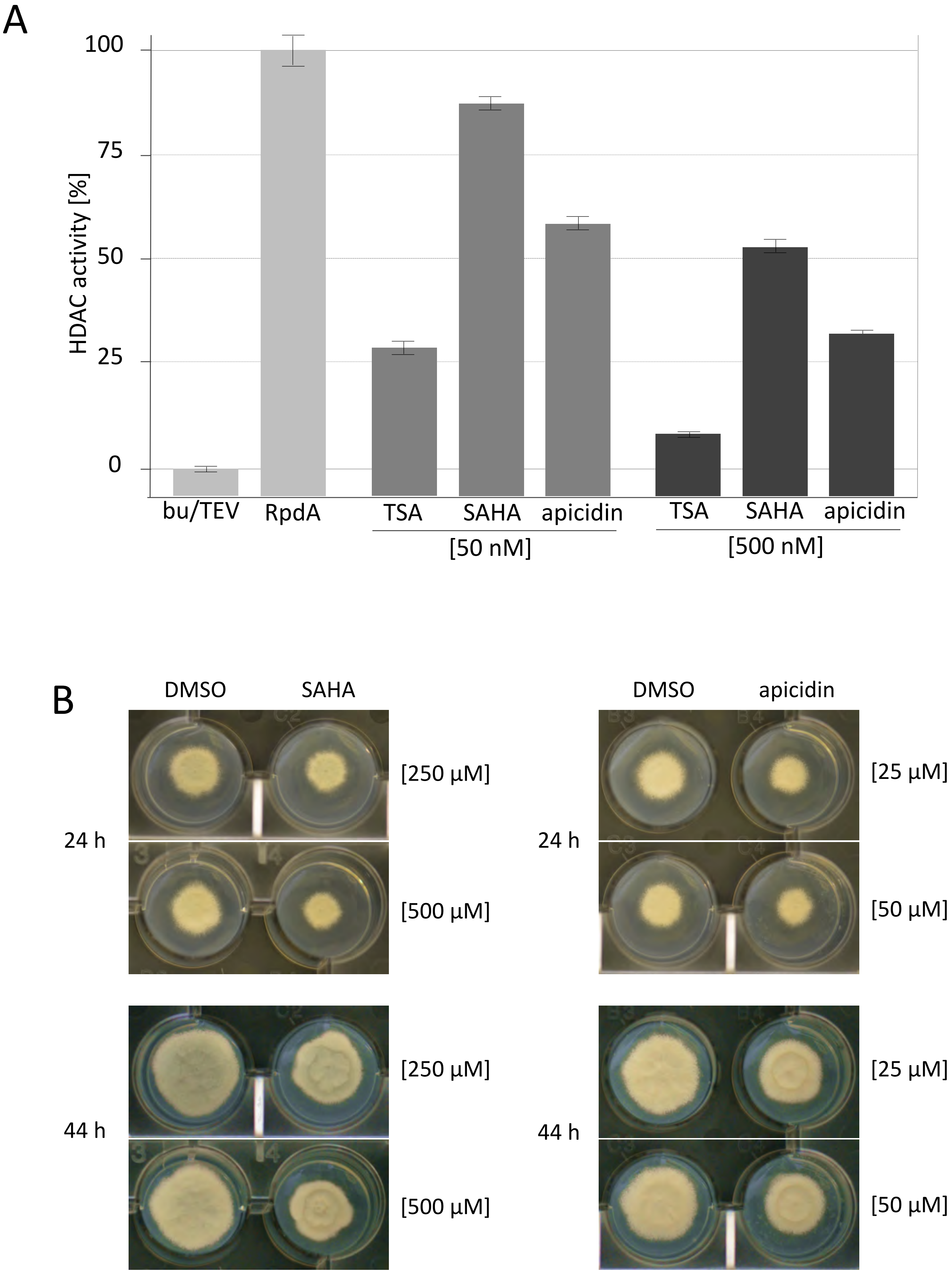

Figure S4 – Efficacy of vorinostat (SAHA) and apicidin against RpdA activity in comparison to TSA (A) and the effect on mycelial growth of *Aspergillus fumigatus* (B).
