# Supplementary material for: A Class 1 Histone Deacetylase with Potential as an Antifungal Target
Source: mBio. 2016 Nov 1;7(6):e00831-16. doi: 10.1128/mBio.00831-16 (PMC5090035; doi:10.1128/mBio.00831-16)
Supplement: Figure S5 — Localization of the expressed RpdA C terminus and of RpdA with deletion of charged C-terminal regions C12 and C6, respectively. Venus-tagged RpdA variants were expressed under the control of xylPp in strain TSG5 comprising mRFP-tagged H2A under the control of the gpdA promoter. For microscopic analysis, strains were grown on coverglasses in eight-well plates under xylPp inductive conditions. Hyphae were viewed under a light microscope (LM) and also, for subcellular localization of the expression products, examined by confocal laser scanning microscopy at a magnification of ×630. Nuclei (H2A-mRFP) are red, and the distribution of expressed Venus-tagged RpdA variants del-C6 and del-C12 and the RpdA C terminus (C-Ter) is shown in green (RpdA-Venus). Download [file mbo005163048sf5.pdf]

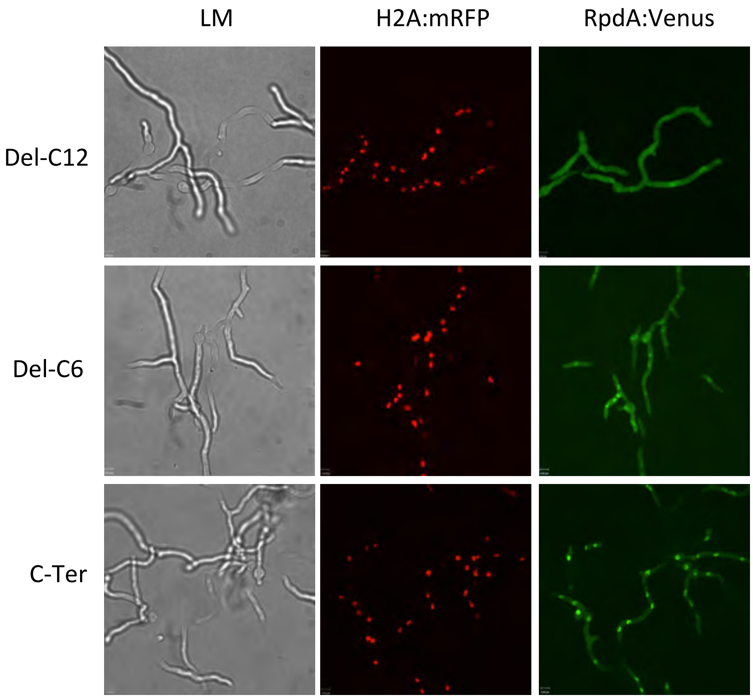

Figure S5 – Localization of the expressed RpdA C-terminus and of RpdA with deletions of the charged C-terminal regions C12 and C6, respectively.
