# Supplementary material for: A Class 1 Histone Deacetylase with Potential as an Antifungal Target
Source: mBio. 2016 Nov 1;7(6):e00831-16. doi: 10.1128/mBio.00831-16 (PMC5090035; doi:10.1128/mBio.00831-16)
Supplement: Figure S6 — Localization of RpdA variants with different deletions within fungus-specific, acidic region C12. Venus-tagged RpdA variants were expressed under the control of xylPp in strain TSG5 comprising mRFP-tagged H2A under the control of the gpdA promoter. For microscopic analysis, strains were grown on coverglasses in eight-well plates under xylPp inductive conditions. Hyphae were viewed under a light microscope (LM) and also, for subcellular localization of the RpdA variants, examined by confocal laser scanning or epifluorescence microscopy (DelE) at a magnification of ×630. Nuclei (H2A-mRFP) are red, and the distribution of expressed Venus-tagged RpdA variants (RpdA-Venus) is shown in green. Download [file mbo005163048sf6.pdf]

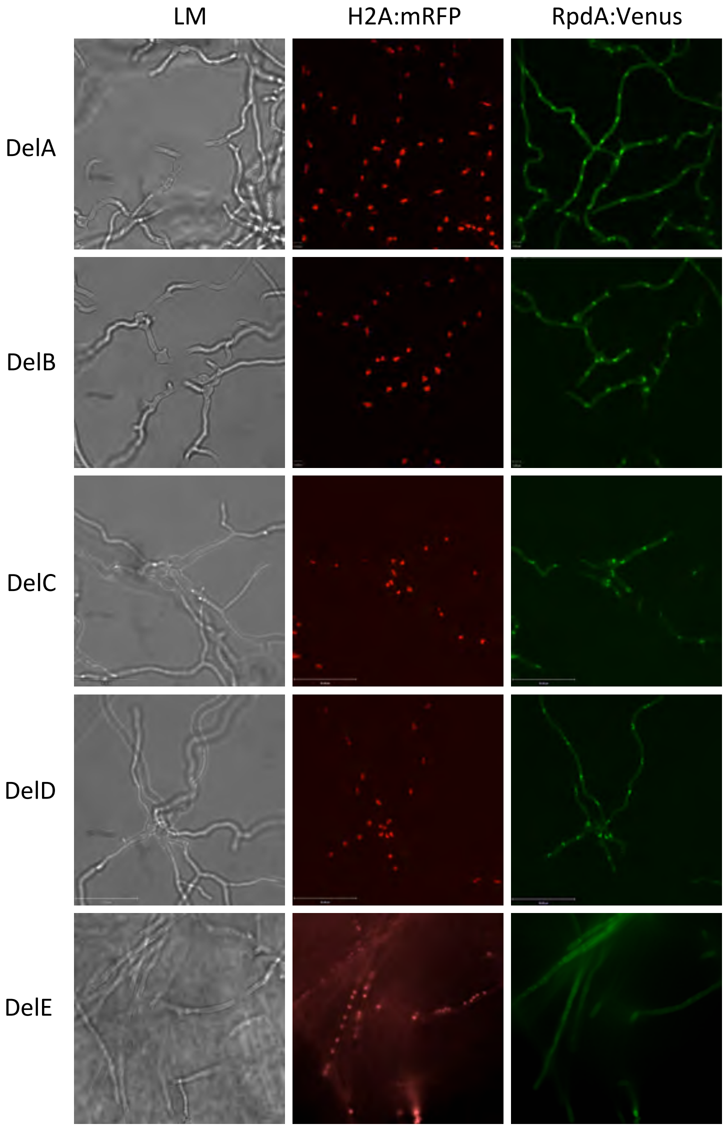

Figure S6 – Localization of RpdA variants with different deletions within the fungal-specific acidic region C12.
