# Supplementary material for: A Class 1 Histone Deacetylase with Potential as an Antifungal Target
Source: mBio. 2016 Nov 1;7(6):e00831-16. doi: 10.1128/mBio.00831-16 (PMC5090035; doi:10.1128/mBio.00831-16)
Supplement: Table S1 — Genotypes of strains used for the expression of RpdA variants (A) and heterologous RPD3-type HDACs (B) or in TSA inhibition and heterokaryon rescue experiments (C). The lab name and the pseudonym used in this report are specified. The genetic characteristic of strains are indicated as rpdAp::pyrG-alcAp-rpdA [alcA(p)-rpdA], which indicates replacement of the endogenous rpdA promoter (rpdAp) with the alcohol dehydrogenase promoter (alcAp). In this case, pyrG was used as an auxotrophic marker for selection of transformants. For expression strains, the corresponding expression plasmids and the selection marker used are shown. The gene in the construct to be expressed under the control of the xylanase promoter (xylPp) of P. chrysogenum is indicated in the fourth column. Strains H4, A18, A89, and RIB211 were used for sexual crosses. Experiments were done with at least three independent transformants of each genotype shown. [file mbo005163048st1.pdf]

**Supplemental Table S1: Genotypes of strains used for the expression of RpdA variants (A), heterologous RPD3-type HDACs (B) or in TSA inhibition and heterokaryon rescue experiments (C).**

## A) *Aspergillus nidulans* strains used for expression of different RpdA-variants

| Strain | Ref. name study | Origin | Variance with regard to RpdA | Genotype | Reference |
|---|---|---|---|---|---|
| A89 | – | FGSC | – | *biA1; argB2; veA1* | – |
| A768 | – | FGSC | – | *pyrG89; riboB2; yA2; chaA1; veA1* | – |
| H4 | H4 | A768 | – | *ΔhdaA::pyrG; pyrG89; riboB2; yA2; chaA1; veA1* | Tribus *et al.*, 2005, Eukaryotic Cell 4:1736-1745 |
| A18 | A18 | A768 | *alcA(p):rpdA* | *rpdAp::pyrG:alcAp:rpdA; pyrG89; riboB2; yA2; chaA1; veA1* | Tribus *et al.*, 2010, Mol Biol Cell 21:345-353 |
| RIB211 | RIB211 | H4 X A18 | *alcA(p):rpdA* | *alcA(p):rpdA; ΔhdaA::pyrG; riboB2; yA2; veA1; PyrG+* | this study |
| RIB214 | RIB214 | RIB211 X A89 | *alcA(p):rpdA* | *alcA(p):rpdA; veA1; argB2; yA2; PyrG+* | – " – |
| TSG5 | TSG5 | RIB214 | *alcA(p):rpdA; H2A:RFP* | *alcA(p):rpdA; veA1; argB2; yA2; PyrG+; pME3857; BleR+* | – " – |
| TIB92n1 | FL:Venus | TSG5 | *alcA(p):rpdA; xylP(p):rpdA:Venus* | *alcA(p):rpdA; veA1; argB2; yA2; pIB92:argB; pME3857; ArgB+; PyrG+; BleR+* | – " – |
| TIB32.1 | FL:TAP | RIB214 | *alcA(p):rpdA; xylP(p):rpdA:TAP* | *alcA(p):rpdA; veA1; argB2; yA2; pIB32:argB; ArgB+; PyrG+* | – " – |
| TSG1 | H158A(:Venus) | TSG5 | *alcA(p):rpdA; xylP(p):rpdAH158A:Venus* | *alcA(p):rpdA; veA1; argB2; yA2; pSG1:argB; pME3857; ArgB+; PyrG+; BleR+* | – " – |
| pSG1_TAP1 | H158A(:TAP) | RIB214 | *alcA(p):rpdA; xylP(p):rpdAH158A:TAP* | *alcA(p):rpdA; veA1; argB2; yA2; pSG1_TAP: argB; ArgB+; PyrG+* | – " – |
| TSG2 | D193A(:Venus) | TSG5 | *alcA(p):rpdA; xylP(p):rpdAD193A:Venus* | *alcA(p):rpdA; veA1; argB2; yA2; pSG2:argB; pME3857; ArgB+; PyrG+; BleR+* | – " – |
| pSG2_TAP2 | D193A(:TAP) | RIB214 | *alcA(p):rpdA; xylP(p):rpdAD193A:TAP* | *alcA(p):rpdA; veA1; argB2; yA2; pSG2_TAP: argB; ArgB+; PyrG+* | – " – |
| NTD4 | Del-N18 | RIB211 | *alcA(p):rpdA; xylP(p):rpdA Δ N18* | *alcA(p):rpdA; ΔhdaA::pyrG; veA1; riboB2; yA2; pXylPRpdA.NTD; RiboB+; PyrG+* | – " – |
| TIB93n1 | Del-C18(:Venus) | TSG5 | *alcA(p):rpdA; xylP(p):rpdA Δ C18:Venus* | *alcA(p):rpdA; veA1; argB2; yA2; pIB93:argB; pME3857; ArgB+; PyrG+; BleR+* | – " – |
| TIB94n1 | C12(:Venus) | TSG5 | *alcA(p):rpdA; xylP(p):rpdA Δ C12:Venus* | *alcA(p):rpdA; veA1; argB2; yA2; pIB94:argB; pME3857; ArgB+; PyrG+; BleR+* | – " – |
| T6_A12 | Ala-C12(:Venus) | TSG5 | *alcA(p):rpdA; xylP(p):rpdAC12:Ala:Venus* | *alcA(p):rpdA; veA1; argB2; yA2; pIB92_TRU6(ALA):argB; pME3857; ArgB+; PyrG+; BleR+* | – " – |
| TIB130.5 | Ala-C12(:TAP) | RIB214 | *alcA(p):rpdA; xylP(p):rpdAC12:Ala:TAP* | *alcA(p):rpdA; veA1; argB2; yA2; pIB130:argB; ArgB+; PyrG+* | – " – |
| TIB95n2 | C6(:Venus) | TSG5 | *alcA(p):rpdA; xylP(p):rpdAC Δ C6:Venus* | *alcA(p):rpdA; veA1; argB2; yA2; pIB95:argB; pME3857; ArgB+; PyrG+; BleR+* | – " – |
| TIB105n1 | DelA(:GFP) | TSG5 | *alcA(p):rpdA; xylP(p):rpdA Δ region A* | *alcA(p):rpdA; veA1; argB2; yA2; pIB105:argB; pME3857; ArgB+; PyrG+; BleR+* | – " – |
| TIB106n2 | DelB(:GFP) | TSG5 | *alcA(p):rpdA; xylP(p):rpdA Δ region B* | *alcA(p):rpdA; veA1; argB2; yA2; pIB106:argB; pME3857; ArgB+; PyrG+; BleR+* | – " – |
| TIB107n2 | DelC(:GFP) | TSG5 | *alcA(p):rpdA; xylP(p):rpdA Δ region C* | *alcA(p):rpdA; veA1; argB2; yA2; pIB107:argB; pME3857; ArgB+; PyrG+; BleR+* | – " – |
| TIB108n3 | DelD(:GFP) | TSG5 | *alcA(p):rpdA; xylP(p):rpdA Δ region D* | *alcA(p):rpdA; veA1; argB2; yA2; pIB108:argB; pME3857; ArgB+; PyrG+; BleR+* | – " – |
| T12_5_2 | DelE(:Venus) | TSG5 | *alcA(p):rpdA; xylP(p):rpdA Δ region E* | *alcA(p):rpdA; veA1; argB2; yA2; pIB92_TRU12:argB; pME3857; ArgB+; PyrG+; BleR+* | – " – |
| AS6_2 | AlaA(:Venus) | TSG5 | *alcA(p):rpdA; xylP(p):rpdAAlaA:Venus* | *alcA(p):rpdA; veA1; argB2; yA2; pIB92_AS6:argB; pME3857; ArgB+; PyrG+; BleR+* | – " – |
| TIB131.9 | AlaA(:TAP) | RIB214 | *alcA(p):rpdA; xylP(p):rpdAAlaA:TAP* | *alcA(p):rpdA; veA1; argB2; yA2; pIB131:argB; ArgB+; PyrG+* | – " – |
| AS6P1_R1 | AlaB(:Venus) | TSG5 | *alcA(p):rpdA; xylP(p):rpdAAlaB:Venus* | *alcA(p):rpdA; veA1; argB2; yA2; pIB92_AS6P1:argB; pME3857; ArgB+; PyrG+; BleR+* | – " – |
| TIB132.2 | AlaB(:TAP) | RIB214 | *alcA(p):rpdA; xylP(p):rpdAAlaB:TAP* | *alcA(p):rpdA; veA1; argB2; yA2; pIB132:argB; ArgB+; PyrG+* | – " – |

## B) *Aspergillus nidulans* strains used for expression of heterologous RPD3-type enzymes

| Strain | Ref. name study | Origin | Variance with regard to class 1-type HDACs | Genotype | Reference |
|---|---|---|---|---|---|
| HDAC1.12 | Hsa | RIB211 | *alcAp:rpdA; xylPp:HDAC1 (human)* | *alcA(p):rpdA; xylPp:HDAC1; pyrG89;riboB2; yA2; chA1* | this study |
| TIB123.4 | Hsa(:Venus) | RIB211 | *alcAp:rpdA; xylPp:HDAC1:Venus (human)* | *alcA(p):rpdA; pIB123:argB; pyrG89; riboB2; yA2; chaA1* | – " – |
| HDC2.2.6 | Cca | RIB211 | *alcAp:rpdA; xylPp:HDC2 (C. carbonum)* | *alcA(p):rpdA; xylPp:HDC2; hdaA::pyrG; pyrG89;riboB2; yA2; chA1* | – " – |
| TIB115n2 | Pch(:Venus) | TSG5 | *alcAp:rpdA; H2A:RFP; xylPp:RpdAPch:Venus (P. chrysogenum)* | *alcA(p):rpdA; veA1; argB2; yA2; pIB115:argB; pME3857; ArgB+; PyrG+; BleR+* | – " – |
| TIB110n3 | Ncr(:Venus) | TSG5 | *alcAp:rpdA; H2A:RFP; xylPp:RpdANcr:Venus (N. crassa)* | *alcA(p):rpdA; veA1; argB2; yA2; pIB110:argB; pME3857; ArgB+; PyrG+; BleR+* | – " – |

## C) Strains used for the heterokaryon rescue experiment (A1280) and TSA-inhibition (all others)

| Strain | Origin | Genotype | Reference |
|---|---|---|---|
| ***Aspergillus fumigatus*** | | | |
| A1163 | FGSC | wt | – |
| A1280 | FGSC | *akuA::loxP* | – |
| ***Aspergillus terreus*** | | | |
| T9 | – | wt | Maurer *et al.*, 2015, Virulence 6:591-598 |
| ***Neurospora crassa*** | | | |
| FGSC #4200 | FGSC | wt | – |
| ***Penicillium chrysogenum*** | | | |
| Q176 (ATCC 10002) | – | wt | Kaiserer *et al.*, 2003, Arch Micribiol 180:204-210 |
